# Biosynthetic Gene Cluster Mining and In Vitro Evaluation of the Antimicrobial and Cytotoxic Potential of *Xenorhabdus thuongxuanensis*

**DOI:** 10.3390/biology15191716

**Published:** 2026-09-28

**Authors:** Yaowapa Sikhaophong, Sophit Khanthawong, Jatuporn Ngoenkam, Sutthirat Sitthisak, Apichat Vitta, Liam K. R. Sharkey, Sacha J. Pidot, Aunchalee Thanwisai

**Affiliations:** 1 Department of Microbiology and Parasitology, Faculty of Medical Science, Naresuan University, Phitsanulok 65000, Thailand; yaowapas67@nu.ac.th (Y.S.); sophitth@nu.ac.th (S.K.); jatupornn@nu.ac.th (J.N.); sutthirats@nu.ac.th (S.S.); apichatv@nu.ac.th (A.V.); 2 Department of Microbiology and Immunology, Doherty Institute, 792 Elizabeth Street, Melbourne 3000, Australia; liam.sharkey@unimelb.edu.au (L.K.R.S.); sacha.pidot@unimelb.edu.au (S.J.P.)

**Keywords:** *Xenorhabdus thuongxuanensis*, biosynthetic gene clusters (BGCs), genomic characteristics, bioactive secondary metabolites, natural products

## Abstract

*Xenorhabdus* species are prolific producers of diverse bioactive natural products. However, the genomic characteristics, biosynthetic potential, and biological activities of *Xenorhabdus thuongxuanensis* remain poorly characterized. This study aimed to characterize the genomic features and biosynthetic potential of *X. thuongxuanensis* strains KLK17.5 and KLK18.1 through comparative genomic analyses and to evaluate the antibacterial, antifungal, and cytotoxic activities of their crude extracts. Both strains had genome sizes and GC contents comparable to those of other *Xenorhabdus* species, with average nucleotide identity (ANI) values of 96–98% supporting their classification as *X. thuongxuanensis*. Functional annotation identified genes involved in secondary metabolism, while antiSMASH predicted diverse biosynthetic regions showing similarity to known BGCs, including those associated with holomycin, putrebactin, avaroferrin, and aryl polyenes. The crude extracts exhibited antibacterial activity against several antibiotic-resistant bacteria with minimum inhibitory concentration (MIC) values ranging from 0.2 to 6.25 mg/mL. They also inhibited *Candida albicans* and *Cryptococcus neoformans*, with inhibition zones ranging from 7.5 to 12 mm and from 10 to 42.5 mm, respectively. The crude extract from KLK18.1 exhibited cytotoxicity against HepG2 cells, with an IC_50_ value of 10.85 µg/mL. These findings expand the current knowledge of the genomic, biosynthetic, and biological potential of *X. thuongxuanensis* and provide a basis for future characterization of its BGC-associated metabolites.

## Simple Summary

*Xenorhabdus* bacteria are a significant source of natural compounds with diverse biological activities, including antimicrobial and cytotoxic effects. However, some species within the genus *Xenorhabdus* remain poorly understood in terms of their genomic characteristics, biosynthetic potential, and biological activities. In this study, we investigated the genomes of two *Xenorhabdus thuongxuanensis* strains, KLK17.5 and KLK18.1, and compared them with those of related *Xenorhabdus* species to better understand their biosynthetic potential. We also evaluated the antibacterial, antifungal, and cytotoxic activities of crude extracts from both strains. Our results revealed genes involved in secondary metabolism and diverse predicted biosynthetic regions, some of which showed similarity to known biosynthetic gene clusters (BGCs). The crude extracts exhibited antibacterial and antifungal activities, while the KLK18.1 extract demonstrated cytotoxicity against HepG2 cells. These findings expand our understanding of the genomic characteristics, biosynthetic potential, and biological activities of *X. thuongxuanensis* and provide a foundation for future studies of its bioactive metabolites.

## 1. Introduction

*Xenorhabdus thuongxuanensis* is a Gram-negative bacterium belonging to the family *Morganellaceae,* which forms a mutualistic association with entomopathogenic nematodes of the genus *Steinernema*. Members of the genus *Xenorhabdus* are recognized as important producers of structurally diverse natural products with a broad range of biological activities. These natural products exhibit a wide spectrum of biological activities, including antimicrobial, insecticidal, nematicidal, antiprotozoal, and cytotoxic activities [1,2]. Many natural products produced by *Xenorhabdus* species are synthesized through BGCs. These clusters contain genes encoding the enzymes required for secondary metabolite biosynthesis. Variation in BGC composition among *Xenorhabdus* species and strains contributes to the remarkable diversity of their secondary metabolites and reflects differences in their biosynthetic potential. Major BGC classes identified in *Xenorhabdus* include nonribosomal peptide synthetases (NRPSs), polyketide synthases (PKSs), and hybrid NRPS–PKS pathways [3,4,5,6].

Representative metabolites include GameXPeptides [7,8,9], xenematide [10], rhabduscin [11], xenocoumacins [12,13,14,15,16,17], and fabclavines [13,18,19,20,21]. To date, several BGCs responsible for the biosynthesis of *Xenorhabdus* secondary metabolites have been experimentally characterized, providing direct evidence linking specific BGCs to the production of bioactive compounds. Among these, the xenocoumacin BGC (*xcn*) [15] and the fabclavine BGC (*fcl*) [21] are well-characterized examples demonstrating this relationship. These experimentally characterized BGCs highlight the value of BGC analysis for predicting the biosynthetic potential of *Xenorhabdus* genomes and provide a framework for interpreting experimentally observed biological activities.

Comparative genomic analysis, including pan-genome analysis and functional classification based on clusters of orthologous groups (COGs), provides an effective framework for investigating genomic diversity and biosynthetic potential among *Xenorhabdus* species. These approaches facilitate the assessment of BGC diversity and the identification of genomic features potentially associated with secondary metabolite biosynthesis. Although an increasing number of *Xenorhabdus* genomes have become available and the genome sequence of *X. thuongxuanensis* has been reported, this species was initially described primarily through taxonomic characterization using a polyphasic approach encompassing phylogenetic/genomic, phenotypic, biochemical, and chemotaxonomic analyses [22]. Its secondary metabolic potential and biological activities, however, were not investigated in that study.

The secondary metabolic potential of *X. thuongxuanensis* is therefore of particular interest, especially with respect to its antimicrobial activity. Antimicrobial resistance (AMR) remains a major global challenge, and the genetic plasticity of bacteria facilitates their adaptation and acquisition of diverse resistance mechanisms [23]. Therefore, antibiotic-resistant bacteria represent relevant test organisms for evaluating the antibacterial potential of crude extracts from *X. thuongxuanensis*. In addition, pathogenic fungi associated with invasive fungal infections (IFIs), such as *Candida albicans* and *Cryptococcus neoformans*, which cause invasive candidiasis and cryptococcosis, respectively, remain important human pathogens [24]. Furthermore, assessing cytotoxic activity against cancer cells provides a broader assessment of the biological potential of the bacterial extracts.

Therefore, this study aimed to characterize the genomes of two *X. thuongxuanensis* strains in Thailand and investigate their biosynthetic potential through comparative genomic analyses of *Xenorhabdus* species, including pan-genome analysis and COG-based functional classification. In addition, the BGCs of the two isolates were identified and compared with those of publicly available *X. thuongxuanensis* genomes. Finally, the antibacterial, antifungal, and cytotoxic activities of crude extracts from the two isolates were evaluated to further assess their biological potential.

## 2. Materials and Methods

### 2.1. Bacterial Strains and Genome Sequencing

Bacterial strains KLK17.5 and KLK18.1 were associated with an entomopathogenic nematode isolated from soil samples in Khlong Lan District, Kamphaeng Phet Province, Thailand, in 2023. In addition, both bacterial strains were identified as *Xenorhabdus thuongxuanensis* based on analysis of the *recA* gene using primers *recA*_F (5′-GCT ATT GAT GAA AAT AAA CA-3′) and *recA*_R (5′-RAT TTT RTC WCC RTT RTA GCT-3′) [25]. Genomic DNA was extracted using the Presto™ Mini gDNA Bacteria Kit (Geneaid Biotech Ltd., New Taipei City, Taiwan). Whole-genome sequencing was performed using the Illumina NextSeq 500 and Oxford Nanopore Technologies platforms, following the methodology described in references [26,27].

### 2.2. Genome Assembly and Annotation

Raw sequencing data were initially subjected to quality assessment using FastQC version 0.12.1 [28]. Adapter sequences and low-quality reads were subsequently removed using Trimmomatic version 0.36 [29] for short-read data, while long-read sequences were filtered using Filtlong version 0.3.1 [30]. The filtered reads were processed through hybrid genome assembly using Unicycler version 0.5.1 [31], which integrates both short- and long-read data. To further improve assembly continuity and reduce graph complexity, an additional round of assembly refinement was performed using the original short-read dataset. Assembly quality was evaluated using QUAST version 5.3.0 [32,33]. Genome annotation was carried out using Prokka version 1.14.6 [34], and genome visualization was performed using Proksee (build 25 February 2026) [35].

### 2.3. Comparative Genomic Analysis

To investigate genomic similarity and taxonomic relationships, ANI was calculated using fastANI version 1.3 [36] to compare KLK17.5 and KLK18.1 with 34 reference *Xenorhabdus* genomes listed in Table S1. Pairwise comparisons were performed between each isolate and the reference genomes, using an ANI threshold of ≥95% for species-level delineation. Genome completeness and contamination were assessed using CheckM2 version 1.1.0 [37] for all 36 genomes included in the comparative genomic analysis. Gene functional analysis was performed using anvi’o version 9 [38], with further data processing performed using Python version 3.13.0 and pandas version 3.0.1. Pan-genome analysis and visualization of gene presence–absence patterns were conducted at the genus level using anvi’o version 9 [38]. Amino acid sequence similarities were calculated using DIAMOND version 2.2.0, and gene clusters were identified using the Markov Cluster Algorithm (MCL) version 22-282, with a minimum bit-score (minbit) heuristic of 0.5 and an MCL inflation parameter of 2.0.

### 2.4. Prediction of BGCs

The BGC predictions were generated using antiSMASH version 8.0 [39], including KnownClusterBlast and the MIBiG database [40]. Emphasis was placed on nonribosomal peptide synthetase (NRPS) and polyketide synthase (PKS) clusters, including associated tailoring enzymes and transporter genes. Enzyme active sites were predicted using ActiveSiteFinder and further verified through BLASTp (version 2.17.0+, accessed on 10 May 2026) searches against homologous proteins and comparison with BGCs and functional annotations previously reported in the literature [27,41].

### 2.5. Antibacterial Activity of Crude Extracts of X. thuongxuanensis Against Antibiotic-Resistant Bacteria

#### 2.5.1. Preparation of Crude Extracts

Bacterial cultures were initially grown on nutrient bromothymol blue agar (NBTA) (HiMedia Laboratories, Thane, India) at 28 °C for 48 h. A single colony was then inoculated into 6 mL of LB broth and incubated at 28 °C with shaking at 200 rpm for 20 h to obtain a starter culture. Subsequently, the starter culture was transferred into 600 mL of Luria–Bertani (LB) broth (HiMedia Laboratories, India) in each of three 1000 mL Erlenmeyer flasks. The cultures were incubated at 28 °C under shaking conditions at 200 rpm for 48 h. Following incubation, 1200 mL of ethyl acetate was added to each flask, the contents were mixed thoroughly, and the flasks were left at room temperature for 24 h to allow phase separation. The upper ethyl acetate phase (approximately 800 mL) was collected and evaporated using a rotary evaporator (Buchi, Switzerland). This extraction and evaporation process was repeated three times for each flask. The dried crude extract was weighed to determine the extract yield and then dissolved in dimethyl sulfoxide (DMSO) to obtain a concentration of 500 mg/mL. Finally, the crude extract solution was transferred into sterile 1.5 mL microcentrifuge tubes and stored at −20 °C for further antibacterial activity assays [42,43,44].

#### 2.5.2. Preparation of Antibiotic-Resistant Bacterial Cultures

Antibiotic-resistant bacterial strains were obtained from the Department of Microbiology, Naresuan University, Phitsanulok, Thailand. The bacterial strains used in this study included *Escherichia coli* ATCC 35218 (extended-spectrum β-lactamase [ESBL]-producing), PB1 (ESBL-producing and multidrug-resistant [MDR]), PB30 (MDR), and PB231 (ESBL-producing and carbapenem-resistant Enterobacterales [CRE]); *Klebsiella pneumoniae* ATCC 700603 (ESBL-producing), PB5 (ESBL-producing and MDR), and PB21 (ESBL-producing and CRE); *Acinetobacter baumannii* AB320 and AB324 (extensively drug-resistant [XDR]), and AB321 and AB322 (MDR); *Staphylococcus aureus* ATCC 20475, PB36, and PB57 (methicillin-resistant *Staphylococcus aureus* [MRSA]); and *Pseudomonas aeruginosa* ATCC 27853 (MDR). The isolates were initially cultured on LB agar and incubated at 37 °C for 24 h. Subsequently, a single colony from each culture was inoculated into 3 mL of LB broth and incubated at 37 °C with shaking at 180 rpm for 24 h. The cell suspension was then resuspended in 0.85% NaCl, and the turbidity was adjusted to a 0.5 McFarland standard using a turbidity meter (DEN-1B McFarland Densitometer, SIA Biosan, Riga, Latvia) [45].

#### 2.5.3. Antibacterial Activity of Crude Extracts Using the Disk Diffusion Method

A sterile cotton swab was dipped into the antibiotic-resistant bacterial suspension prepared in Section 2.5.2 and evenly spread over the surface of Mueller–Hinton agar (MHA) (HiMedia Laboratories, India) plates. Subsequently, 10 µL of the crude extracts of both *X. thuongxuanensis* strains (100 mg/mL) were applied to 6 mm sterile Antibiotic Assay paper disks (1 mg/disk). The disks were then placed on the MHA plates inoculated with the bacterial suspension. The plates were incubated at 37 °C for 24 h. Inhibition zones were observed and measured, and the results were compared with those obtained with the standard antibiotic (positive control) and DMSO (negative control).

#### 2.5.4. Antibacterial Activity of Crude Extracts Using MIC and Minimum Bactericidal Concentration (MBC) Assays

Crude extracts that showed inhibition zones in the disk diffusion assay were further evaluated for their minimum inhibitory concentrations (MICs) using the broth microdilution method. Briefly, 100 µL of Mueller–Hinton broth (MHB) (HiMedia Laboratories, India) was dispensed into each well of a 96-well microtiter plate. Crude extracts of *X. thuongxuanensis* (100 mg/mL) were added to the first row and subjected to two-fold serial dilution to obtain final concentrations ranging from 50 to 0.02 mg/mL (rows 1–9). Antibiotic-resistant bacterial cultures were prepared as described in Section 2.5.2 and diluted to approximately 5 × 10^5^ CFU/mL in MHB. Subsequently, 100 µL of the bacterial suspension was added to the wells containing the test extracts and to the control wells (rows 1–11). Row 10 served as the bacterial growth control and contained the bacterial suspension without extract treatment, whereas row 11 served as the DMSO solvent control and contained the bacterial suspension with the corresponding concentration of DMSO. Row 12, containing MHB alone, was included as the sterility control. All experiments were performed duplicate, and the plates were incubated at 37 °C for 24 h. Bacterial growth was assessed based on visible turbidity. The MIC was defined as the lowest concentration of the extract that completely inhibited visible bacterial growth. For MBC determination, 10 µL aliquots from the wells containing bacterial suspensions (rows 1–11) were spotted onto Mueller–Hinton agar (MHA) plates and allowed to air-dry at room temperature. The plates were then incubated at 37 °C for 24 h. The MBC was defined as the lowest concentration of the extract that resulted in no visible bacterial growth on the MHA plates [46].

#### 2.5.5. Time-Kill Assay

The *S. aureus* PB57 (MRSA), *K. pneumoniae* PB5 (ESBL-producing/MDR), and *P. aeruginosa* ATCC 27853 were cultured according to the procedure described in Section 2.5.2 and diluted to approximately 5 × 10^5^ CFU/mL in MHB. The cultures were then exposed to crude extracts at a 2X MIC for *S. aureus* PB57 (MRSA) and *K. pneumoniae* PB5 (ESBL+MDR), and at 1X MIC for *P. aeruginosa* ATCC 27853 (MDR), with the corresponding concentrations prepared using MHB. The assays were conducted in 1.5 mL microcentrifuge tubes by adding 100 µL each of the antibiotic-resistant bacterial suspension and the crude extract to each tube. The incubation time points were set at 0, 0.5, 1, 2, 4, 8, 12, 16, 20, and 24 h. Following incubation, samples were subjected to ten-fold serial dilutions using 0.85% NaCl solution. Subsequently, a 10-μL aliquot of each dilution was spotted onto MHA plates (in duplicate) and incubated at 37 °C for 24 h. Following incubation, the colonies were counted. The three antibiotic-resistant bacterial isolates were tested in triplicate, and statistical differences were analyzed using GraphPad Prism 10 software.

### 2.6. Antifungal Activity of Crude Extracts of X. thuongxuanensis Using the Disk Diffusion Method

The *Candida albicans* ATCC 90028 and *Cryptococcus neoformans* strains, which were isolated from the environment and provided by the Department of Microbiology and Parasitology, Faculty of Medical Science, Naresuan University, Thailand, were cultured on Potato Dextrose Agar (PDA) (HiMedia Laboratories, India). The concentrations of the cultures were subsequently adjusted to a 0.5 McFarland standard using 0.85% NaCl and evenly spread over the surface of PDA plates. Subsequently, 10 µL of the crude extracts of both *X. thuongxuanensis* strains (100 mg/mL) was applied to 6 mm sterile antibiotic assay paper disks (1 mg/disk). The disks were then placed on the PDA plates inoculated with the fungal suspension. The plates were incubated at 37 °C for 24–48 h. Inhibition zones were observed and measured, and the results were compared with those obtained with the standard antifungal agent (positive control) and DMSO (negative control).

### 2.7. The 3-(4,5-Dimethylthiazol-2-yl)-2,5-diphenyl-2H-tetrazolium Bromide (MTT) Assay

#### 2.7.1. Cell Culture

Human hepatocellular carcinoma cells (HepG2) were cultured in Dulbecco’s Modified Eagle Medium (DMEM; HiMedia Laboratories, India) supplemented with 10% fetal bovine serum (FBS) and 1% penicillin–streptomycin. Cells were maintained in 35 cm^2^ culture flasks and incubated at 37 °C in a humidified atmosphere containing 5% CO_2_ until they reached approximately 80–90% confluence.

#### 2.7.2. Cytotoxicity of Crude Extracts From *X. thuongxuanensis* Against HepG2 Cells Using the MTT Assay

HepG2 cells were harvested by trypsinization using trypsin–EDTA and resuspended in complete DMEM. Cells were seeded into 96-well plates at a density of 1 × 10^4^ cells per well (100 µL) and incubated for 24 h. Crude extracts from both *X. thuongxuanensis* strains were prepared at a concentration of 200 µg/mL and added to the test wells (100 µL per well) to achieve a final concentration of 100 µg/mL. Each treatment was performed in at least five replicates. Cells treated with 0.5% DMSO served as the negative control, while DMEM alone was used as the blank control. After 24 h of incubation, cells were washed with PBS and incubated with fresh DMEM (200 µL) containing MTT reagent. Plates were then incubated for 3 h to allow the formation of formazan crystal formation. The medium was subsequently removed, and the formazan crystals were dissolved in DMSO (200 µL per well). Absorbance was measured at 540 nm using a Synergy HT microplate reader (BioTek Instruments, Inc., Winooski, VT, USA). Isolates showing promising cytotoxic activity were further evaluated using a range of concentrations (100, 50, 25, 12.5, 6.25, 3.13, and 1.56 µg/mL). Experimental procedures were performed as described above. Each condition was tested in at least five technical replicates and three independent biological replicates. IC_50_ values were calculated using GraphPad Prism version 10 (GraphPad Software, LLC, Boston, MA, USA) based on dose–response curves.

## 3. Results

### 3.1. Genome Characteristics and Comparative Genomic Analysis

The *Xenorhabdus* isolates examined in this study were obtained from *Steinernema* sp. recovered from soil samples collected in Thailand. KLK17.5 and KLK18.1 were identified as *X. thuongxuanensis* based on 99.85% sequence similarity of the *recA* gene. Sequencing revealed genome sizes for both strains of 4.17 Mb for KLK17.5 (accession number SAMN56417105) and 3.80 Mb for KLK18.1 (accession number SAMN56417106). The genome assembly of KLK17.5 consisted of 13 contigs, with the largest contig measuring 1.71 Mb and an N50 value of 1.68 Mb. In comparison, the genome assembly of KLK18.1 consisted of 12 contigs, with both the largest contig and N50 measuring 3.57 Mb. The GC content of the KLK17.5 and KLK18.1 genomes was 43.16% and 42.82%, respectively. The numbers of coding sequences (CDSs) were predicted as 3712 and 3424, respectively.

For comparison with previously available *X. thuongxuanensis* genomes, strains CIP111194^T^ (accession number SAMEA117660565) and 30TX1 (accession number SAMN05375129) were included. Their genome sizes were 3.87 and 3.93 Mb, respectively; their G+C contents were 43.21% and 43.04%, respectively; and the numbers of predicted CDSs were 3405 and 3471, respectively. Overall, the four *X. thuongxuanensis* strains showed comparable genome sizes and G+C contents. The numbers of tmRNA genes were similar among the four strains, whereas slight differences were observed in the numbers of tRNA and rRNA genes. The genomic characteristics of the four *X. thuongxuanensis* strains are summarized in Table S2. Circular genome maps of KLK17.5 and KLK18.1 showing the distribution of CDSs, tRNA genes, rRNA genes, G+C content, and GC skew are presented in Figure 1.

The ANI analysis was conducted to compare the genomes of 36 strains belonging to the genus *Xenorhabdus*, representing 33 species. The ANI values ranged from 81% to 100%, as shown in Table S3. The ANI values of KLK17.5 and KLK18.1 compared with previously available *X. thuongxuanensis* genomes ranged from 96% to 98%. KLK17.5 showed ANI values of 97.61% and 97.63% with *X. thuongxuanensis* CIP111194ᵀ and 30TX1, respectively, whereas KLK18.1 showed corresponding ANI values of 96.72% and 96.61%. These values were above the commonly accepted ANI threshold for species delineation, supporting the assignment of KLK17.5 and KLK18.1 to *X. thuongxuanensis*. Genome quality assessment using CheckM2 showed estimated genome completeness values of 99.80–100% across the 36 genomes included in the pan-genome analysis. Thirty-two genomes had estimated contamination levels ranging from 0% to 4%, whereas four genomes showed higher contamination estimates ranging from 6.59% to 13.21%. For the newly sequenced strains, read-mapping analysis showed mapping rates of 96.85% and 96.87% for KLK17.5 and KLK18.1, respectively. The corresponding genome coverage values were 99.89% and 99.97%, with mean sequencing depths of 164.80× and 196.18×, respectively (Table S1).

Pan-genome analysis of the 36 genomes identified a total of 143,226 genes, which were clustered into 14,253 orthologous gene groups. These included 67,778 core genes assigned to 1754 core orthologous gene groups, 69,115 accessory genes assigned to 6421 accessory orthologous gene groups, and 6333 unique genes assigned to 6078 unique orthologous gene groups, as shown in Figure 2 and Table S4. Based on the total number of orthologous gene groups, core, accessory, and unique gene groups accounted for 12.31%, 45.05%, and 42.64%, respectively. Gene presence–absence analysis across the 36 genomes showed that core gene clusters were present in all genomes examined, whereas accessory and unique gene clusters showed variable distributions among genomes, as illustrated in Figure S1.

### 3.2. Functional Annotation Based on COG Analysis

Functional annotation based on the COG database was performed for a total of 143,226 genes from 36 strains of the genus *Xenorhabdus*. Among these, 110,347 genes were successfully assigned to COG functional categories, accounting for 77.04% of the total number of genes. The annotated genes were classified into 23 COG functional categories. The most abundant COG categories included Translation, ribosomal structure and biogenesis (J), accounting for 8.5%; Amino acid transport and metabolism (E), accounting for 8.2%; Mobilome: prophages, transposons (X), accounting for 7.5%; Cell wall/membrane/envelope biogenesis (M), accounting for 7.1%; and Transcription (K), accounting for 6.7% of all COG-annotated genes, as shown in Figure 3 and Table S5.

Genes assigned to the COG category Secondary metabolites biosynthesis, transport, and catabolism (Q) accounted for 2.9% of all COG-annotated genes. Based on the anvi’o pan-genome analysis, genes in this category were further classified into core, accessory, and unique genes, accounting for 1.95%, 5.27%, and 5.66%, respectively, as shown in Figure S2 and Table S6. These findings suggest that genes involved in secondary metabolite biosynthesis, transport, and catabolism are distributed across both conserved gene sets shared by all strains and strain-specific gene sets. When considering KLK17.5 and KLK18.1, genes assigned to category Q accounted for 3.04% and 2.92%, respectively. These proportions were marginally lower than those observed in the previously available *X. thuongxuanensis* strains 30TX1 and CIP111194ᵀ, which contained 3.25% and 3.21% of genes assigned to category Q, respectively (Table S7).

### 3.3. Comparative Analysis of BGCs

The presence of BGCs in the genomes of four strains, including *X. thuongxuanensis* KLK17.5, KLK18.1, 30TX1 (reference strain), and CIP111194^T^ (type strain), was analyzed using antiSMASH. Several types of predicted biosynthetic regions were identified, including aryl polyene, NIS siderophore, betalactone, NRPS, butyrolactone, phosphonate, azole-containing RiPP, terpene precursor, CDPS, NRPS-like, and hybrid regions involving NRPS and other BGC types. Several predicted BGC types were shared among the four genomes, although differences in their biosynthetic profiles were evident (Tables S8–S11).

Based on antiSMASH predictions, several regions showed high similarity to known BGCs. NRPS-associated regions showed high similarity to BGCs associated with the biosynthesis of holomycin, rhabduscin, kolossin, and bovienimide A biosynthesis, whereas NIS siderophore regions showed high similarity to BGCs associated with the biosynthesis of putrebactin and avaroferrin. NRPS-like regions showed high similarity to the xenematide BGC, while aryl polyene regions showed high similarity to known aryl polyene BGCs. Strain-specific differences were also observed in the predicted biosynthetic profiles. In KLK17.5, a predicted region showed high similarity to the rhabduscin BGC. In KLK18.1, manual BLASTp analysis showed that a protein within the predicted region shared 68.99% amino acid identity with a protein associated with the bovienimide A BGC. However, this sequence similarity alone does not confirm the production of bovienimide A by KLK18.1. In addition, the previously reported strain 30TX1 contained predicted regions showing high similarity to the xenematide and kolossin BGCs. For the desferrioxamine E-associated regions in strains 30TX1 and CIP111194^T^, manual BLASTp analysis revealed 53.72% amino acid sequence identity to the corresponding reference protein (Figure 4; Tables S8–S12).

### 3.4. Antibacterial Activity of Crude Extracts of X. thuongxuanensis Using the Disk Diffusion Method

Crude extracts from both *X. thuongxuanensis* strains demonstrated broad-spectrum antibacterial activity, inhibiting the growth of 12–15 pathogenic bacterial strains, including both Gram-positive and Gram-negative bacteria. Both extracts exhibited inhibitory activity against *S. aureus* (MRSA) strains, as well as MDR and ESBL-producing bacteria. Notably, the crude extract from strain KLK18.1 inhibited all tested multidrug-resistant bacterial strains and generally produced larger inhibition zones than the crude extract from KLK17.5 strain. These findings indicate that KLK18.1 exhibited stronger and broader antibacterial activity than KLK17.5 against the tested pathogenic bacteria (Table S13 and Figure S3).

### 3.5. Antibacterial Activity of Crude Extracts of X. thuongxuanensis Using MIC and MBC

Both crude extracts of *X. thuongxuanensis* exhibited antimicrobial activity against *S. aureus*, with MIC values ranging from 0.2 to 0.39 mg/mL and MBC values ranging from 0.78 to 6.25 mg/mL. Notably, the KLK18.1 crude extract demonstrated the strongest activity, with an MBC of 0.78 mg/mL against *S. aureus*. Similarly, both extracts showed activity against *K. pneumoniae* PB5, with MIC values of 0.2–0.39 mg/mL and MBC values of 0.78–3.13 mg/mL. Among the two extracts, the KLK18.1 crude extract exhibited an MIC of 0.2 mg/mL and an MBC of 0.78 mg/mL. In addition, the KLK18.1 crude extract exhibited potent activity against *P. aeruginosa* ATCC 27853, with an MIC of 0.2 mg/mL and an MBC of 0.39 mg/mL. Overall, the KLK18.1 crude extract consistently demonstrated lower MIC and MBC values than the KLK17.5 crude extract against the tested multidrug-resistant bacterial strains, indicating greater antibacterial potency. Notably, the KLK18.1 extract exhibited both MIC and MBC values below 1 mg/mL against *S. aureus* strains ATCC 20475, PB36, and PB57, *K. pneumoniae* PB5, and *P. aeruginosa* ATCC 27853 (Table S14). These findings indicate that the KLK18.1 extract demonstrated antibacterial activity against selected multidrug-resistant bacterial pathogens, supporting further investigation of this strain as a potential source of bioactive compounds.

### 3.6. Time–Kill Assay

The antibacterial activity of crude extracts from *X. thuongxuanensis* isolates KLK17.5 and KLK18.1 was evaluated against *P. aeruginosa* ATCC 27853, *S. aureus* PB57, and *K. pneumoniae* PB5 using the time–kill assay. The crude extracts were tested at 1× MIC against *P. aeruginosa* ATCC 27853 and at 2× MIC against *S. aureus* PB57 and *K. pneumoniae* PB5. Bacterial counts were monitored as log_10_ CFU/mL over 24 h and compared with the untreated control, as shown in Figure 5. Two-way repeated-measures ANOVA revealed significant effects of time, treatment, and the time × treatment interaction on bacterial counts (*p* < 0.0001). For *P. aeruginosa* ATCC 27853, Tukey’s multiple comparisons test showed no significant difference in bacterial counts between the KLK17.5 and KLK18.1 crude extract treatments at any time point. Nevertheless, both treatment groups exhibited significantly lower bacterial counts than the untreated control from 1 h onward, with no significant difference observed at 0.5 h. Furthermore, no colonies of *P. aeruginosa* ATCC 27853 were detected between 8 and 24 h under the plating conditions used. In contrast, strain-dependent differences were observed for *K. pneumoniae* PB5 and *S. aureus* PB57. The KLK18.1 crude extract resulted in significantly lower bacterial counts than the KLK17.5 extract from 4 to 24 h (*p* < 0.0001). The KLK17.5 extract, however, showed a more limited reduction in bacterial counts, with significant differences from the untreated control observed only at selected time points.

Overall, the two crude extracts exhibited comparable antibacterial activity against *P. aeruginosa* ATCC 27853, whereas KLK18.1 demonstrated greater and more sustained bactericidal activity against *K. pneumoniae* PB5 and *S. aureus* PB57, as reflected by the consistently lower bacterial counts during the 24-h assay period (Figure 5).

### 3.7. Antifungal Activity of Crude Extracts of X. thuongxuanensis Using the Disk Diffusion Method

Crude extracts from both *X. thuongxuanensis* strains exhibited antifungal activity against *C. albicans*, producing inhibition zones of 7.5 mm and 12 mm for KLK17.5 and KLK18.1, respectively. Against *C. neoformans*, KLK17.5 and KLK18.1 produced inhibition zones of 10 mm and 42.5 mm, respectively. The inhibition zone produced by KLK18.1 against *C. neoformans* was larger than that of the fluconazole control under the same experimental conditions. Fluconazole produced inhibition zones of 36 mm against *C. neoformans* and 37.9 mm against *C. albicans*. However, inhibition-zone diameters cannot be used to directly compare the antifungal potency of the crude extracts with that of fluconazole because differences in compound composition and diffusion through the agar can influence the observed inhibition zones.

### 3.8. Cytotoxic Activity of Crude Extracts of X. thuongxuanensis Against HepG2 Cells Using the MTT Assay

The cytotoxic activity of crude extracts from both *X. thuongxuanensis* strains was evaluated against HepG2 human hepatocellular carcinoma cells using the MTT assay. Among the tested extracts, the crude extract from KLK18.1 exhibited greater cytotoxic activity, reducing cell viability to 5.08%, whereas cells treated with the KLK17.5 crude extract retained 89.99% viability after 24 h of treatment at 100 µg/mL. Based on this initial screening, the KLK18.1 crude extract was further evaluated over a concentration range of 1.56–100 µg/mL. A concentration-dependent reduction in HepG2 cell viability was observed after 24 h of exposure, with cell viability decreasing below 50% at concentrations of ≥12.5 µg/mL. Nonlinear regression analysis of the concentration–response curve estimated an IC_50_ value of 10.85 µg/mL for the KLK18.1 crude extract (Figure 6).

## 4. Discussion

Genome characterization of KLK17.5 and KLK18.1 was generally comparable to that of the previously available *X. thuongxuanensis* strains 30TX1 and CIP111194ᵀ. The genome sizes of these four strains ranged from 3.7 to 4.2 Mb, with G+C contents of approximately 42–43%. These values were within the range observed among the 36 *Xenorhabdus* genomes analyzed in this study, which showed genome sizes ranging from 3.7 to 5.1 Mb and G+C contents of approximately 42–46%. These findings indicate that KLK17.5 and KLK18.1 share general genomic characteristics with previously available *X. thuongxuanensis* genomes and fall within the genomic ranges observed across the genus *Xenorhabdus*. KLK17.5 and KLK18.1 showed ANI values of 96–98% when compared with *X. thuongxuanensis* strains 30TX1 and CIP111194ᵀ. These ANI values exceeded the commonly accepted species delineation threshold of approximately 95–96% [47], supporting the assignment of KLK17.5 and KLK18.1 to *X. thuongxuanensis*.

Although the genomes of KLK17.5 and KLK18.1 were obtained as draft assemblies consisting of 13 and 12 contigs, respectively, CheckM2 analysis indicated estimated completeness value of 100% for both KLK17.5 and KLK18.1, with estimated contamination levels of 2.95% and 0.47%, respectively. Thus, the assemblies were used for comparative genomic analyses. Nevertheless, as both genomes were draft assemblies, assembly fragmentation may affect the completeness of BGCs located near contig boundaries. Pan-genome analysis of 36 *Xenorhabdus* genomes showed that the number of genes per genome ranged from 3222 in *X. japonica* to 4835 in *X. entomophaga*. A total of 143,226 genes were grouped into 14,253 gene clusters, including 1754 core gene clusters (12.31%), 6421 accessory gene clusters (45.05%), and 6078 unique gene clusters (42.64%). These proportions were consistent with those reported by Awori et al. [48], who analyzed the pan-genome of 27 *Xenorhabdus* genomes and identified 13,469 gene clusters, including 1654 core gene clusters (12.3%), 5992 accessory gene clusters (44.5%), and 5820 strain-specific gene clusters (43%). Taken together, these findings indicate a relatively high proportion of accessory and unique gene clusters among the *Xenorhabdus* genomes examined, reflecting substantial gene-content diversity within the analyzed dataset. However, these proportions should be interpreted with caution because differences in genome assembly quality, including elevated contamination estimates in a small number of genomes, may influence estimates of accessory and unique gene content.

COG analysis showed that 110,347 genes, accounting for 77.04% of the total genes, could be assigned to COG functional categories. The most abundant categories included Translation, ribosomal structure and biogenesis (J) and Amino acid transport and metabolism (E). These results indicate that *Xenorhabdus* genomes contain a high proportion of genes involved in essential cellular processes and amino acid metabolism. This is consistent with the biological characteristics of this bacterial group, which lives in association with nematodes and insect hosts and possesses the potential to produce bioactive compounds [27,49,50].

In addition, genes assigned to COG category Q (Secondary metabolite biosynthesis, transport, and catabolism) accounted for approximately 3% of COG-annotated genes across the four *X. thuongxuanensis* genomes. Although slight differences were observed among the isolates and publicly available genomes (2.92–3.25%), the overall proportions remained consistent, suggesting broadly similar representation of genes assigned to secondary metabolite-related functions within the species. Nevertheless, as the genomes of KLK17.5 and KLK18.1 were draft assemblies consisting of 13 and 12 contigs, respectively, assembly fragmentation may affect the completeness of BGCs located near contig boundaries. Therefore, the predicted BGC profiles should be interpreted with this limitation in mind. Further analysis using antiSMASH revealed variation in the predicted biosynthetic profiles of KLK17.5, KLK18.1, the type strain, and strain 30TX1, including regions showing similarity to known BGCs associated with desferrioxamine E [51], trichrysobactin, cyclic/trichrysobactin/chrysobactin/dichrysobactin [52], aryl polyenes [53], holomycin [54], GameXPeptide C [55], HTTPCA/prepiscibactin/piscibactin [56], kolossin [57], putrebactin, and avaroferrin [58], IOC [9], xenematide [6], bovienimide A [59], rhabduscin [11,60], and xenoamicin A-B [5]. A predicted region showing similarity to the known bovienimide A gene cluster was detected in KLK18.1 but not in KLK17.5. However, the compound(s) responsible for the observed antifungal activity remain unknown, and a direct association between this predicted BGC and the antifungal activity cannot be established based on the present data. Bovienimide A has been proposed as a potentially novel secondary metabolite associated with antifungal activity in *P. vancouverensis* [61].

Furthermore, the detection of a BGC showing similarity to the xenoamicin A–B biosynthetic cluster may also be associated with the biological activity of the crude extract. Xenoamicin has previously been reported to exhibit antiprotozoal and cytotoxic activities and may act through disruption of the cell membrane [5]. Although plasmid-associated signals were detected in KLK17.5 by some prediction tools, MOB-recon and BLASTn analyses supported a chromosomal rather than plasmid-associated location for the examined strain-specific BGC. Nevertheless, because the genomes were obtained as draft assemblies, its genomic localization should be interpreted cautiously.

Regarding antimicrobial activity, the crude extract from KLK18.1 showed MIC values against several multidrug-resistant bacterial strains, mostly around 0.2 mg/mL, and MBC values mostly around 0.78 mg/mL. The time–kill assay further supported the ability of the KLK18.1 crude extract to reduce the bacterial counts of *P. aeruginosa* ATCC 27853 at 0.2 mg/mL and those of *K. pneumoniae* PB5 and *S. aureus* PB57 at 0.4 mg/mL under the tested conditions. The extract also showed inhibitory activity against *C. neoformans* and *C. albicans* in the disk diffusion assay at 1 mg/disk. These findings indicate that the KLK18.1 crude extract demonstrated inhibitory activity against multiple microbial groups under the tested conditions.

However, comparison with the cytotoxicity results requires careful interpretation. The crude extract from KLK18.1 reduced HepG2 cell viability at concentrations of 12.5 µg/mL and above, with cell viability decreasing to 5.08% at 100 µg/mL. Because non-cancerous cells were not evaluated, the selectivity of the extract could not be determined; therefore, the observed effect should be interpreted as cytotoxicity toward HepG2 cells rather than selective anticancer activity. In contrast, a previous study reported that an extract from *Photorhabdus akhurstii* subsp. *akhurstii* (bNN168.5_TH) exhibited activity against antibiotic-resistant bacteria at 0.98 mg/mL without affecting HepG2 cell viability [42]. This finding highlights the importance of cytotoxicity assessment when evaluating antimicrobial potential, as promising antimicrobial candidates should demonstrate effective activity against target microorganisms while exhibiting low toxicity toward non-target mammalian cells [62,63]. Therefore, further evaluation of the KLK18.1 crude extract using non-cancerous cell lines, along with determination of the selectivity index, is required to assess its selectivity and cytotoxicity profile. Although the crude extract from KLK18.1 demonstrated stronger inhibitory activity against the tested antibiotic-resistant bacteria and fungi than the extract from KLK17.5 in several assays, the two isolates differed in their predicted biosynthetic profiles. However, the presence of BGCs in the genome represents biosynthetic potential and does not necessarily indicate actual expression or the production of the corresponding metabolites. Differences in BGC regulation and condition-dependent expression may contribute to variations in metabolite production between strains. Additionally, extraction yield, metabolite stability, and the integrity and functionality of biosynthetic pathways may influence the biological activity observed in crude extracts. Thus, the biological activities of KLK17.5 and KLK18.1 cannot be directly explained by the number or types of predicted BGCs. Further studies, including transcriptomic and metabolomic analyses, as well as bioassay-guided fractionation, are required to identify the factors and metabolites responsible for the differences in biological activity between the two strains.

## 5. Conclusions

In summary, genomic analysis of *Xenorhabdus thuongxuanensis* strain KLK17.5 and KLK18.1 demonstrated that both isolates shared genomic characteristics consistent with previously available *X. thuongxuanensis* genomes. CheckM2 analysis indicated high estimated completeness and low estimated contamination for both genome assemblies. ANI values above the species delineation threshold further supported their classification as *X. thuongxuanensis.* Pan-genome and COG analyses revealed substantial gene-content diversity across the *Xenorhabdus* genomes examined, along with differences in the distribution of functional genes, including those assigned to secondary metabolite biosynthesis, transport, and catabolism. Additionally, antiSMASH-based BGC prediction identified several BGC classes, particularly NRPSs and hybrid BGCs involving NRPS and other biosynthetic types, highlighting the diverse biosynthetic potential of these isolates.

The crude extract from KLK18.1 showed stronger inhibitory activity against the tested antibiotic-resistant bacteria and fungi than the extract from KLK17.5 in several assays, despite KLK17.5 containing a slightly greater number of predicted BGCs. However, the specific BGCs or metabolites responsible for the observed biological activities remain uncertain and require further investigation. The crude extract from KLK18.1 also exhibited cytotoxicity against HepG2 cells. Because non-cancerous mammalian cells were not evaluated, the selectivity of the extract could not be determined. Future studies should include appropriate non-cancerous cell controls, determination of the selectivity index, and isolation and structural characterization of BGC-associated metabolites to clarify their relationships with the predicted BGCs and assess their biological activities.

## Figures and Tables

**Figure 1:**
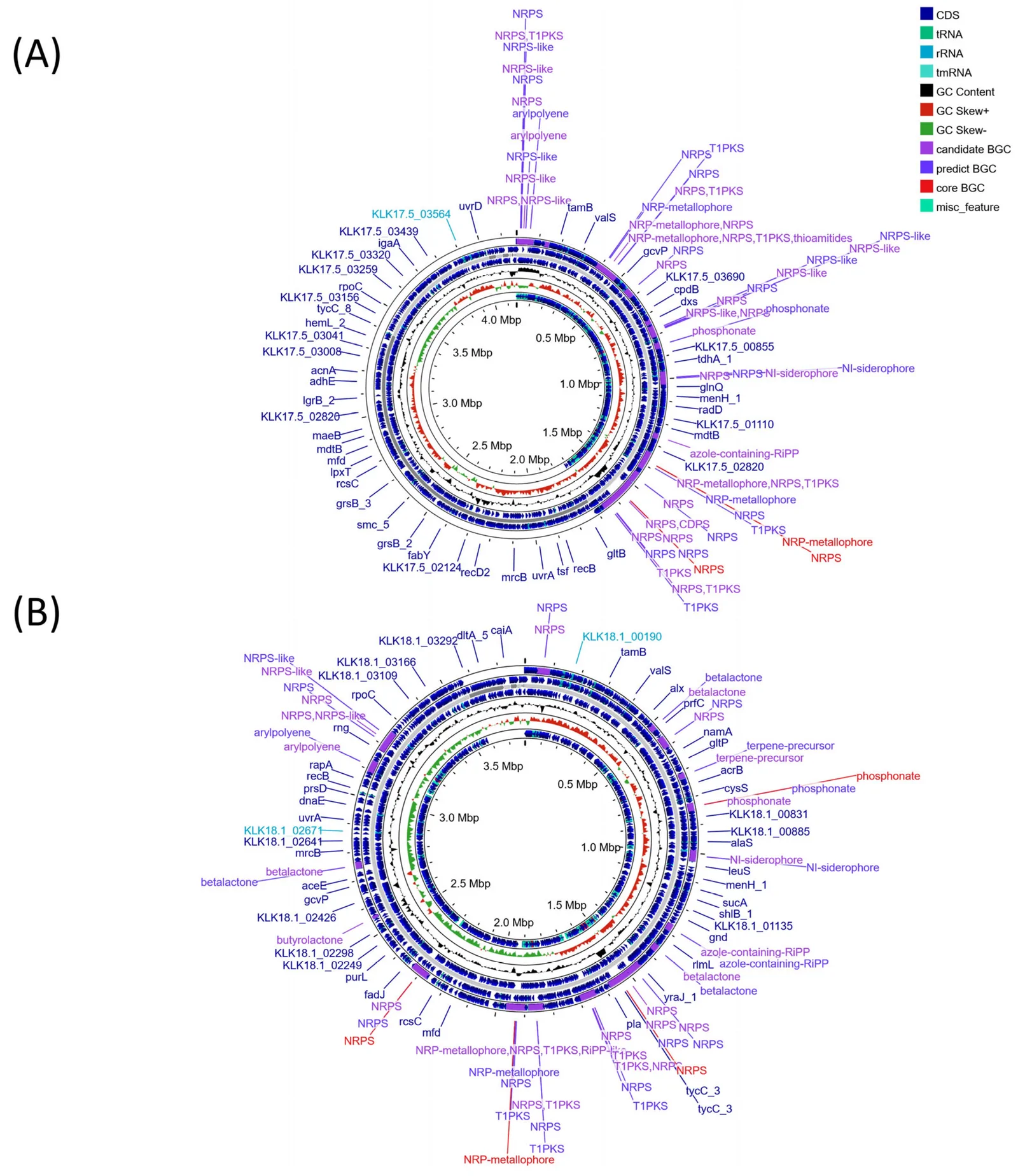
Genome maps of *X. thuongxuanensis* strains KLK17.5 (**A**) and KLK18.1 (**B**). Genomic features include CDS, tRNA, rRNA, mRNA, GC content, GC skew, and BGC-related regions predicted by antiSMASH, including predicted BGC regions, core biosynthetic regions, and candidate BGCs, together with miscellaneous features.

**Figure 2:**
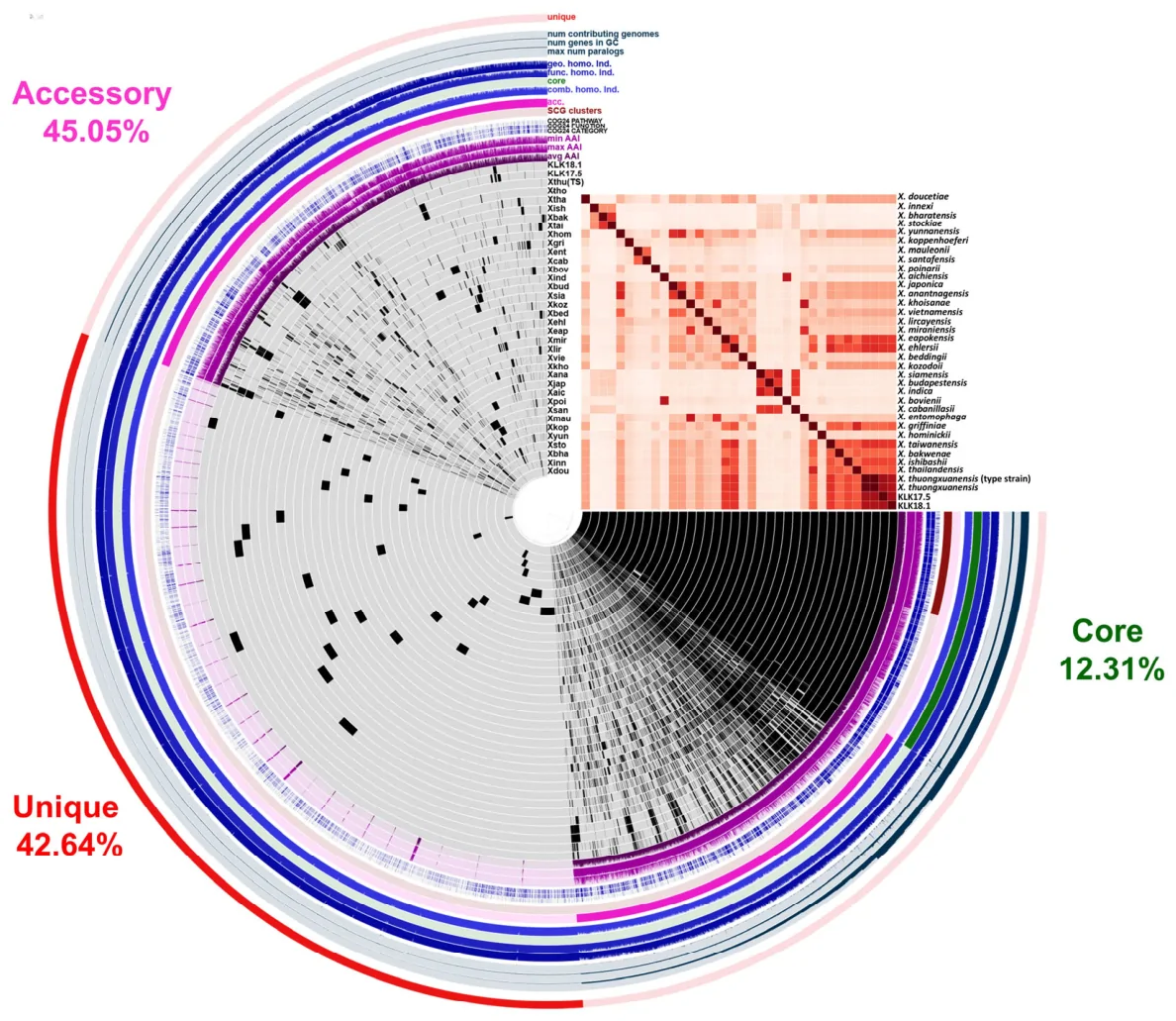
Pan-genome visualization of 36 *Xenorhabdus* genomes. The circular pan-genome plot shows the distribution of gene clusters across the analyzed genomes. Black blocks indicate the presence of genes or gene clusters, whereas light-colored or blank regions indicate their absence in the corresponding genomes. Gene clusters are classified as core, accessory, and unique gene clusters, represented by green, pink, and red, respectively. The heatmap shows pairwise ANI values among all genomes included in the analysis.

**Figure 3:**
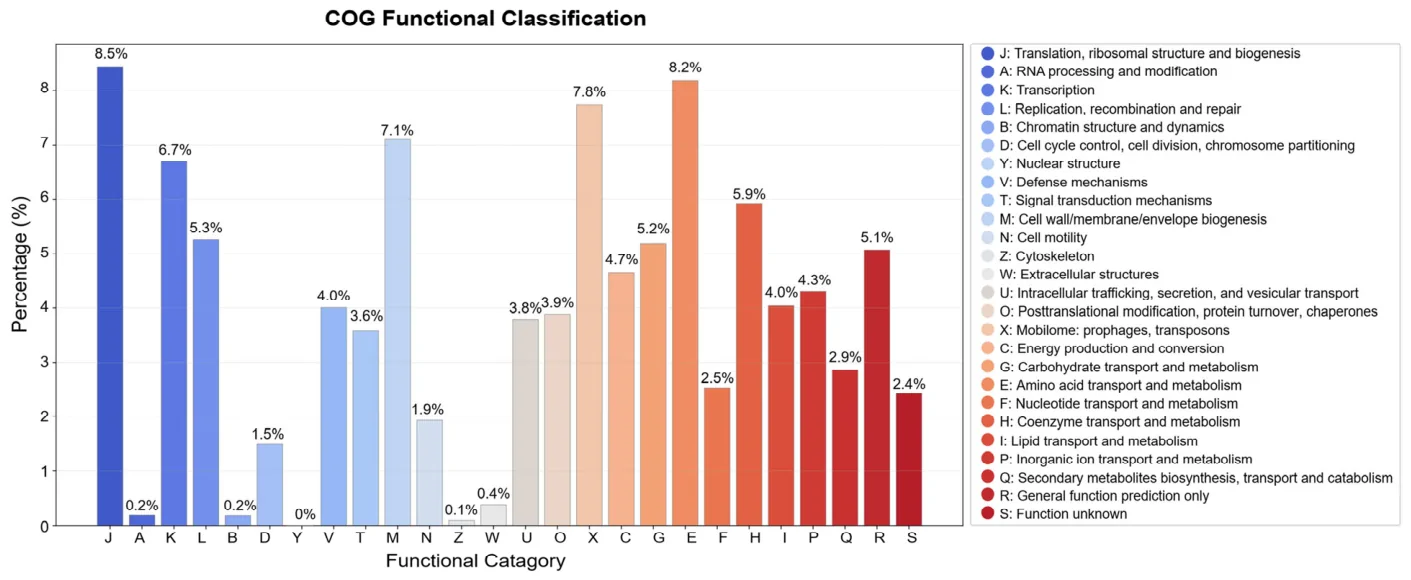
Percentage distribution of COG functional categories in *Xenorhabdus* genomes. The plot shows the percentage distribution of genes assigned to COG functional categories across the analyzed strains.

**Figure 4:**
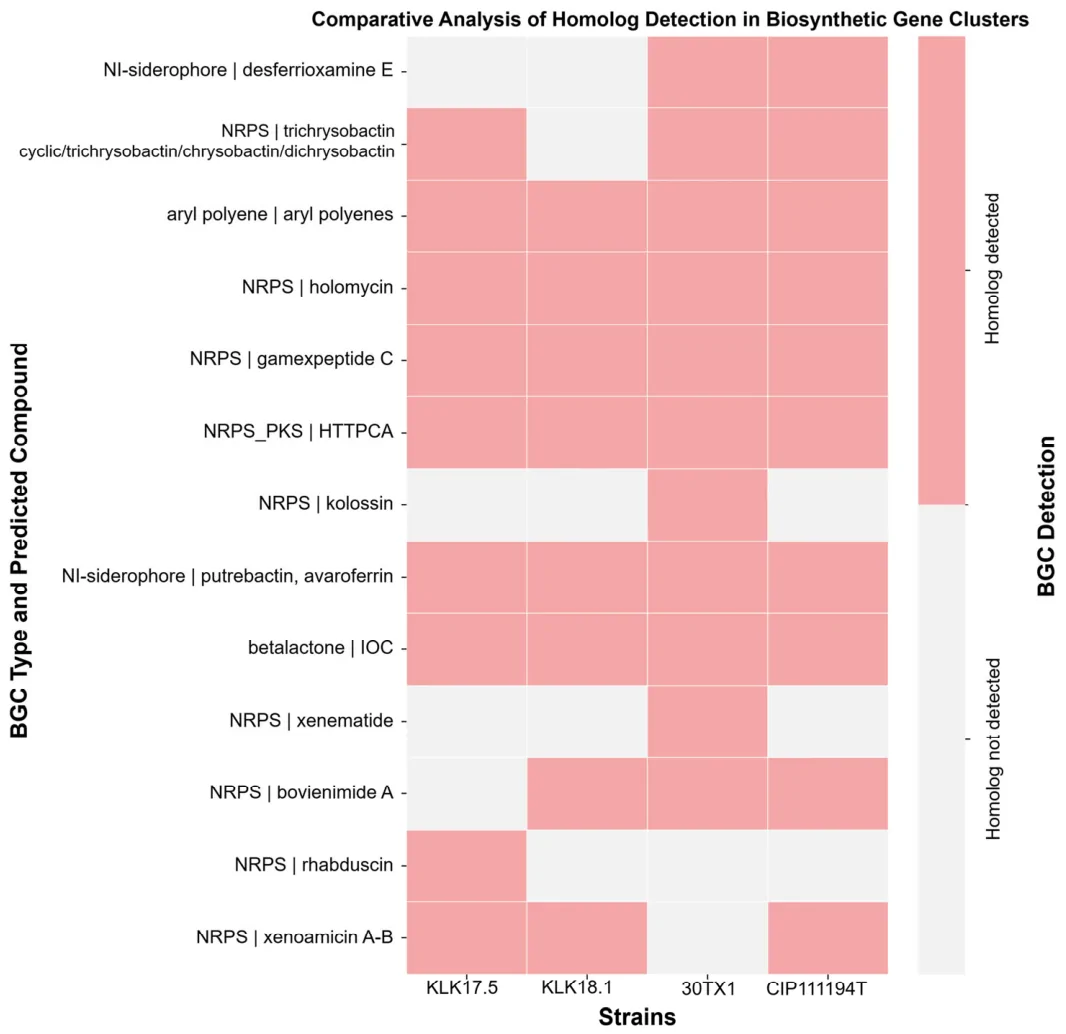
Predicted BGC types and their similarity to known BGCs associated with specific compounds in four *X. thuongxuanensis* genomes.

**Figure 5:**
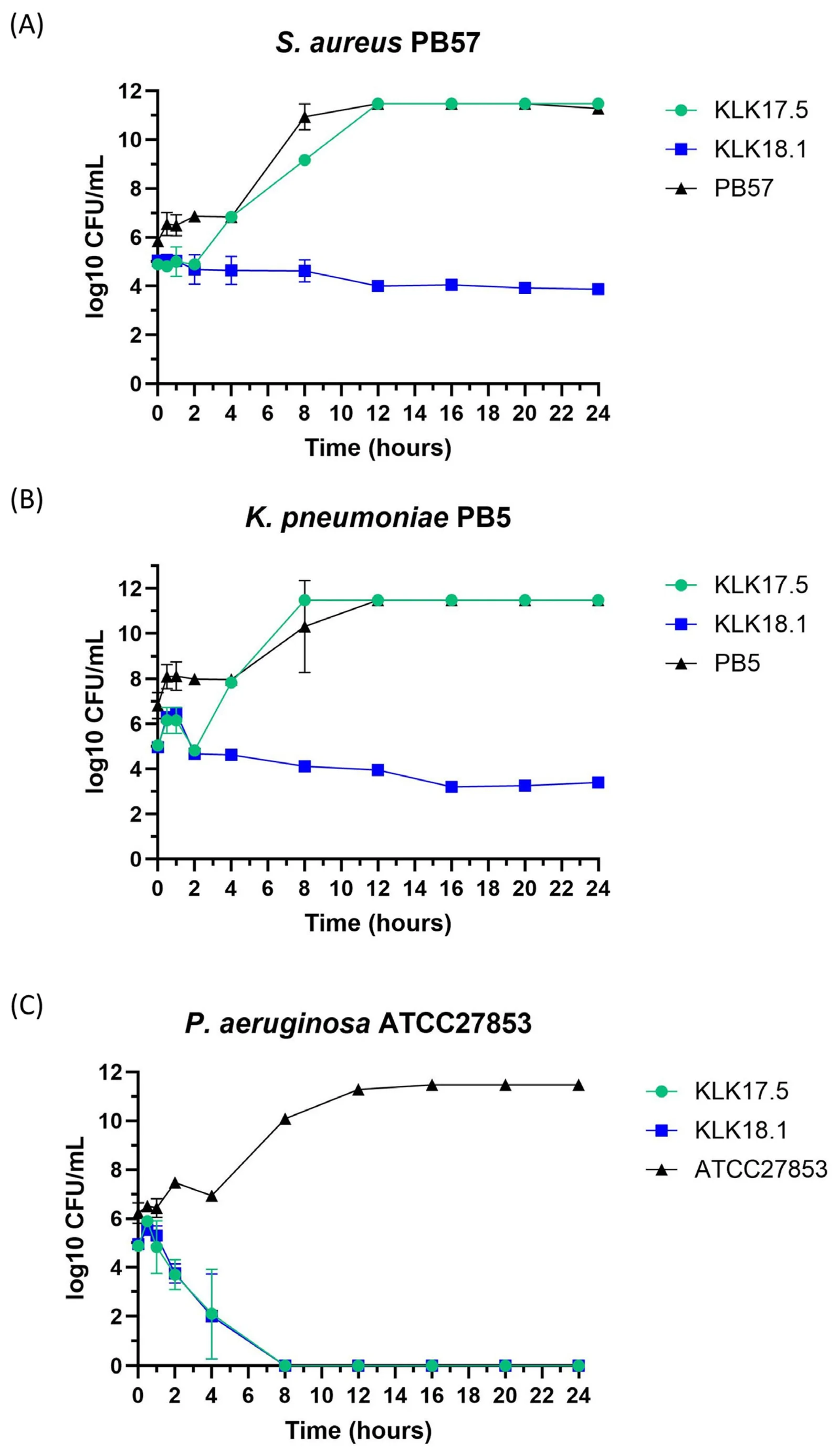
Time–kill assay of crude extracts from *X. thuongxuanensis* strains KLK17.5 and KLK18.1 against *S. aureus* PB57 (MRSA; (**A**)), *K. pneumoniae* PB5 (ESBL+MDR; (**B**)), and *P. aeruginosa* ATCC 27853 (MDR; (**C**)), showing changes in bacterial counts (log_10_ CFU/mL) after exposure to crude extracts at 0, 0.5, 1, 2, 4, 8, 12, 16, 20, and 24 h. The green, blue, and black lines represent treatment with crude extracts from KLK17.5, treatment with crude extracts from KLK18.1, and the untreated control, respectively.

**Figure 6:**
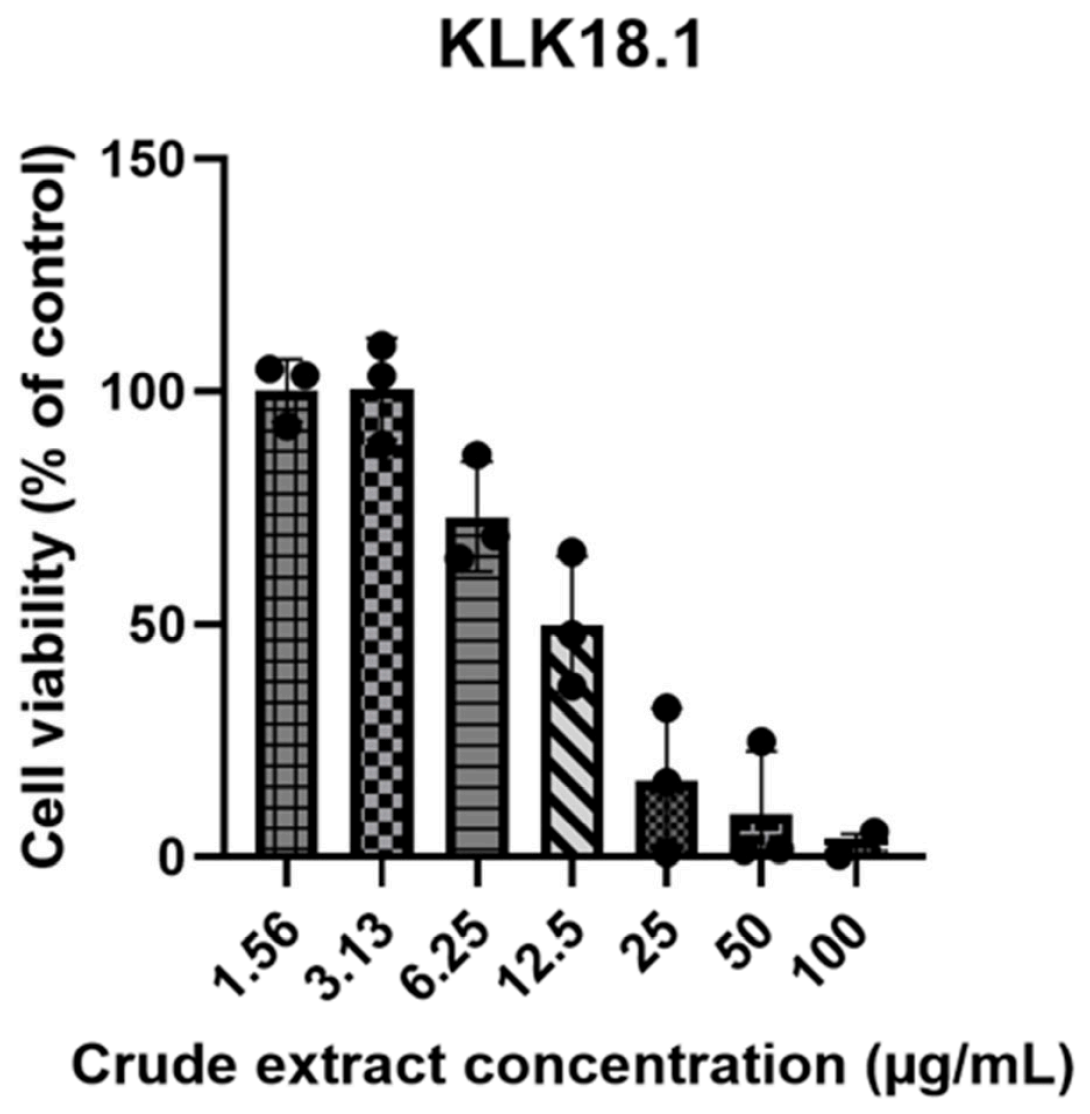
Dose-dependent effects of crude extracts from *X. thuongxuanensis* strain KLK18.1 on cell viability and growth inhibition of HepG2 cells at various concentrations (100–1.56 µg/mL) after 24 h of treatment.

## Data Availability

All relevant data are within the article and its supporting information files.

## Supplementary Materials

The following supporting information can be downloaded at: https://www.mdpi.com/article/10.3390/biology15191716/s1, Figure S1: Gene presence–absence profile across 36 *Xenorhabdus* genomes. The plot shows the distribution of genes across 36 *Xenorhabdus* genomes. Light red indicates gene presence in the corresponding genome, whereas white indicates gene absence; Figure S2: Distribution of COG functional categories among core, accessory, and unique genes; Figure S3: Antibacterial activity of crude extracts from *X. thuongxuanensis* KLK17.5 (1) and *X. thuongxuanensis* KLK18.1 (1), compared with DMSO (N) and an antibiotic disc (P), against pathogenic bacteria including *A. baumannii* AB320 (XDR; A), *A. baumannii* AB321 (MDR; B), *A. baumannii* AB324 (XDR; C), *E. coli* ATCC35218 (ESBL; D), *S. aureus* ATCC20475 (MRSA; E), *S. aureus* PB36 (MRSA; F), *S. aureus* PB57 (MRSA; G), *K. pneumoniae* ATCC700603 (MDR; H), and *K. pneumoniae* PB21 (ESBL+CRE; I); Table S1: Genome characteristics of 34 *Xenorhabdus* species genomes; Table S2: Genomic characteristics of *X. thuongxuanensis* strains assembled in the current study; Table S3: Pairwise comparison of Average Nucleotide Identity (ANI) values among *Xenorhabdus* genomes; Table S4: Pan-genome gene categories across 36 *Xenorhabdus* species genomes; Table S5: Distribution of Clusters of Orthologous Groups (COG) functional classifications across 36 *Xenorhabdus* species genomes; Table S6: Distribution of COG-annotated pan-genome gene categories across COG functional classifications in 36 *Xenorhabdus* species genomes; Table S7. Distribution of Clusters of Orthologous Groups (COG) functional classifications across individual *Xenorhabdus* genomes; Table S8: Predicted biosynthetic gene clusters (BGCs) in *X. thuongxuanensis* KLK17.5 identified using antiSMASH; Table S9: Predicted biosynthetic gene clusters (BGCs) in *X. thuongxuanensis* KLK18.1 identified using antiSMASH; Table S10: Predicted biosynthetic gene clusters (BGCs) in *X. thuongxuanensis* 30TX1 (reference strain) identified using antiSMASH; Table S11: Predicted biosynthetic gene clusters (BGCs) in *X. thuongxuanensis* CIP111194^T^ (type strain) identified using antiSMASH; Table S12: Comparison of annotated features of predicted biosynthetic gene clusters across four *Xenorhabdus thuongxuanensis* strains. Table S13: Antibacterial activity of crude extracts from *X. thuongxuanensis* against drug-resistant bacteria using disc diffusion; Table S14: Antibacterial activity of crude extracts from *X. thuongxuanensis* against drug-resistant bacteria using broth dilution assay.

## Abbreviations

The following abbreviations are used in this manuscript:

BGCs	Biosynthetic Gene Clusters
ANI	Average Nucleotide Identity
COG	Clusters of Orthologous Genes
MIC	Minimum Inhibitory Concentration
MBC	Minimum Bactericidal Concentration
ESBL	extended-spectrum β-lactamase
MDR	multidrug-resistant
CRE	carbapenem-resistant
XDR	extensively drug-resistant
MRSA	methicillin-resistant

## References

[1] Bode H.B. (2009). Entomopathogenic Bacteria as a Source of Secondary Metabolites. Curr. Opin. Chem. Biol..

[2] Webster J., Chen G., Hu K., Li J., Gaugler R. (2002). Entomopathogenic Nematology.

[3] Dreyer J., Malan A.P., Dicks L.M. (2018). Bacteria of the Genus *Xenorhabdus*, a Novel Source of Bioactive Compounds. Front. Microbiol..

[4] Lang G., Kalvelage T., Peters A., Wiese J., Imhoff J.F. (2008). Linear and Cyclic Peptides from the Entomopathogenic Bacterium *Xenorhabdus nematophilus*. J. Nat. Prod..

[5] Zhou Q., Grundmann F., Kaiser M., Schiell M., Gaudriault S., Batzer A., Kurz M., Bode H.B. (2013). Structure and Biosynthesis of Xenoamicins from Entomopathogenic *Xenorhabdus*. Chem.–A Eur. J..

[6] Tobias N.J., Wolff H., Djahanschiri B., Grundmann F., Kronenwerth M., Shi Y.-M., Simonyi S., Grün P., Shapiro-Ilan D., Pidot S.J. (2017). Natural Product Diversity Associated with the Nematode Symbionts *Photorhabdus* and *Xenorhabdus*. Nat. Microbiol..

[7] Bode H.B., Reimer D., Fuchs S.W., Kirchner F., Dauth C., Kegler C., Lorenzen W., Brachmann A.O., Grün P. (2012). Determination of the Absolute Configuration of Peptide Natural Products by Using Stable Isotope Labeling and Mass Spectrometry. Chem.–A Eur. J..

[8] Nollmann F.I., Dauth C., Mulley G., Kegler C., Kaiser M., Waterfield N.R., Bode H.B. (2015). Insect-specific Production of New GameXPeptides in *Photorhabdus luminescens* TTO1, Widespread Natural Products in Entomopathogenic Bacteria. ChemBioChem.

[9] Shi Y.-M., Hirschmann M., Shi Y.-N., Ahmed S., Abebew D., Tobias N.J., Grün P., Crames J.J., Pöschel L., Kuttenlochner W. (2022). Global Analysis of Biosynthetic Gene Clusters Reveals Conserved and Unique Natural Products in Entomopathogenic Nematode-Symbiotic Bacteria. Nat. Chem..

[10] Crawford J.M., Portmann C., Kontnik R., Walsh C.T., Clardy J. (2011). NRPS Substrate Promiscuity Diversifies the Xenematides. Org. Lett..

[11] Crawford J.M., Portmann C., Zhang X., Roeffaers M.B., Clardy J. (2012). Small Molecule Perimeter Defense in Entomopathogenic Bacteria. Proc. Natl. Acad. Sci. USA.

[12] Abebew D., Sayedain F.S., Bode E., Bode H.B. (2022). Uncovering Nematicidal Natural Products from *Xenorhabdus* Bacteria. J. Agric. Food Chem..

[13] Gulsen S.H., Tileklioglu E., Bode E., Cimen H., Ertabaklar H., Ulug D., Ertug S., Wenski S.L., Touray M., Hazir C. (2022). Antiprotozoal Activity of Different *Xenorhabdus* and *Photorhabdus* Bacterial Secondary Metabolites and Identification of Bioactive Compounds Using the easyPACId Approach. Sci. Rep..

[14] Incedayi G., Cimen H., Ulug D., Touray M., Bode E., Bode H.B., Orenlili Yaylagul E., Hazir S., Cakmak I. (2021). Relative Potency of a Novel Acaricidal Compound from *Xenorhabdus*, a Bacterial Genus Mutualistically Associated with Entomopathogenic Nematodes. Sci. Rep..

[15] Park D., Ciezki K., Van Der Hoeven R., Singh S., Reimer D., Bode H.B., Forst S. (2009). Genetic Analysis of Xenocoumacin Antibiotic Production in the Mutualistic Bacterium *Xenorhabdus nematophila*. Mol. Microbiol..

[16] Reimer D., Luxenburger E., Brachmann A.O., Bode H.B. (2009). A New Type of Pyrrolidine Biosynthesis Is Involved in the Late Steps of Xenocoumacin Production in *Xenorhabdus nematophila*. ChemBioChem.

[17] Yang X., Qiu D., Yang H., Liu Z., Zeng H., Yuan J. (2011). Antifungal Activity of Xenocoumacin 1 from *Xenorhabdus nematophilus* var. *pekingensis* against *Phytophthora infestans*. World J. Microbiol. Biotechnol..

[18] Fuchs S.W., Grundmann F., Kurz M., Kaiser M., Bode H.B. (2014). Fabclavines: Bioactive Peptide–Polyketide-polyamino Hybrids from *Xenorhabdus*. ChemBioChem.

[19] Kim I.-H., Ensign J., Kim D.-Y., Jung H.-Y., Kim N.-R., Choi B.-H., Park S.-M., Lan Q., Goodman W.G. (2017). Specificity and Putative Mode of Action of a Mosquito Larvicidal Toxin from the Bacterium *Xenorhabdus innexi*. J. Invertebr. Pathol..

[20] Touray M., Ulug D., Gulsen S.H., Cimen H., Hazir C., Bode H.B., Hazir S. (2024). Natural Products from *Xenorhabdus* and *Photorhabdus* Show Promise as Biolarvicides against *Aedes albopictus*. Pest Manag. Sci..

[21] Wenski S.L., Kolbert D., Grammbitter G.L., Bode H.B. (2019). Fabclavine Biosynthesis in *X. szentirmaii*: Shortened Derivatives and Characterization of the Thioester Reductase FclG and the Condensation Domain-like Protein FclL. J. Ind. Microbiol. Biotechnol..

[22] Kämpfer P., Tobias N.J., Ke L.P., Bode H.B., Glaeser S.P. (2017). *Xenorhabdus thuongxuanensis* sp. nov. and *Xenorhabdus eapokensis* sp. nov., Isolated from *Steinernema* Species. Int. J. Syst. Evol. Microbiol..

[23] Galgano M., Pellegrini F., Catalano E., Capozzi L., Del Sambro L., Sposato A., Lucente M.S., Vasinioti V.I., Catella C., Odigie A.E. (2025). Acquired Bacterial Resistance to Antibiotics and Resistance Genes: From Past to Future. Antibiotics.

[24] Miao B., Yao J., Zhang S., Guo X., Geng Y., Gong J. (2026). Public Health Risks of Pathogenic Fungi in China. iFungi.

[25] Tailliez P., Laroui C., Ginibre N., Paule A., Pagès S., Boemare N. (2010). Phylogeny of *Photorhabdus* and *Xenorhabdus* Based on Universally Conserved Protein-Coding Sequences and Implications for the Taxonomy of These Two Genera. Proposal of New Taxa: *X. vietnamensis* sp. nov., *P. luminescens* subsp. *caribbeanensis* subsp. nov., *P. luminescens* subsp. *hainanensis* subsp. nov., *P. temperata* subsp. *khanii* subsp. nov., *P. temperata* subsp. *tasmaniensis* subsp. nov., and the Reclassification of *P. luminescens* subsp. *thracensis* as *P. temperata* subsp. *thracensis* comb. nov. Int. J. Syst. Evol. Microbiol..

[26] Meesil W., Bode H.B., Rückert-Reed C., Shi Y.-M., Pidot S.J., Muangpat P., Rattanarojpong T., Chantratita N., Sitthisak S., Vitta A. (2025). Genomic Analysis for the Identification of Bioactive Compounds in *Xenorhabdus stockiae* strain RT25. 5. Sci. Rep..

[27] Meesil W., Ardpairin J., Sharkey L.K., Pidot S.J., Vitta A., Thanwisai A. (2025). Whole-Genome Sequencing and Biosynthetic Gene Cluster Analysis of Novel Entomopathogenic Bacteria *Xenorhabdus thailandensis* ALN 7.1 and ALN 11.5. Biology.

[28] Andrews S. (2010). FastQC: A Quality Control Tool for High Throughput Sequence Data.

[29] Bolger A.M., Lohse M., Usadel B. (2014). Trimmomatic: A Flexible Trimmer for Illumina Sequence Data. Bioinformatics.

[30] Wick R., Menzel P. (2019). *Github*; Filtlong: Quality Filtering Tool for Long Reads. https://github.com/rrwick/Filtlong.

[31] Wick R.R., Judd L.M., Gorrie C.L., Holt K.E. (2017). Unicycler: Resolving Bacterial Genome Assemblies from Short and Long Sequencing Reads. PLoS Comput. Biol..

[32] Gurevich A., Saveliev V., Vyahhi N., Tesler G. (2013). QUAST: Quality Assessment Tool for Genome Assemblies. Bioinformatics.

[33] Mikheenko A., Prjibelski A., Saveliev V., Antipov D., Gurevich A. (2018). Versatile Genome Assembly Evaluation with QUAST-LG. Bioinformatics.

[34] Seemann T. (2014). Prokka: Rapid Prokaryotic Genome Annotation. Bioinformatics.

[35] Grant J.R., Enns E., Marinier E., Mandal A., Herman E.K., Chen C., Graham M., Van Domselaar G., Stothard P. (2023). Proksee: In-Depth Characterization and Visualization of Bacterial Genomes. Nucleic Acids Res..

[36] Jain C., Rodriguez-R L.M., Phillippy A.M., Konstantinidis K.T., Aluru S. (2018). High Throughput ANI Analysis of 90K Prokaryotic Genomes Reveals Clear Species Boundaries. Nat. Commun..

[37] Chklovski A., Parks D.H., Woodcroft B.J., Tyson G.W. (2023). CheckM2: A Rapid, Scalable and Accurate Tool for Assessing Microbial Genome Quality Using Machine Learning. Nat. Methods.

[38] Eren A.M., Esen Ö.C., Quince C., Vineis J.H., Morrison H.G., Sogin M.L., Delmont T.O. (2015). Anvi’o: An Advanced Analysis and Visualization Platform for ‘omics Data. PeerJ.

[39] Blin K., Shaw S., Vader L., Szenei J., Reitz Z.L., Augustijn H.E., Cediel-Becerra J.D., de Crécy-Lagard V., Koetsier R.A., Williams S.E. (2025). antiSMASH 8.0: Extended Gene Cluster Detection Capabilities and Analyses of Chemistry, Enzymology, and Regulation. Nucleic Acids Res..

[40] Terlouw B.R., Blin K., Navarro-Munoz J.C., Avalon N.E., Chevrette M.G., Egbert S., Lee S., Meijer D., Recchia M.J., Reitz Z.L. (2023). MIBiG 3.0: A Community-Driven Effort to Annotate Experimentally Validated Biosynthetic Gene Clusters. Nucleic Acids Res..

[41] Meesil W., Muangpat P., Sitthisak S., Rattanarojpong T., Chantratita N., Machado R.A., Shi Y.-M., Bode H.B., Vitta A., Thanwisai A. (2023). Genome Mining Reveals Novel Biosynthetic Gene Clusters in Entomopathogenic Bacteria. Sci. Rep..

[42] Muangpat P., Meesil W., Ngoenkam J., Teethaisong Y., Thummeepak R., Sitthisak S., Tandhavanant S., Chantratita N., Bode H.B., Vitta A. (2022). Genome Analysis of Secondary Metabolite-biosynthetic Gene Clusters of *Photorhabdus akhurstii* subsp. *akhurstii* and Its Antibacterial Activity against Antibiotic-Resistant Bacteria. PLoS ONE.

[43] Muangpat P., Suwannaroj M., Yimthin T., Fukruksa C., Sitthisak S., Chantratita N., Vitta A., Thanwisai A. (2020). Antibacterial Activity of *Xenorhabdus* and *Photorhabdus* Isolated from Entomopathogenic Nematodes against Antibiotic-Resistant Bacteria. PLoS ONE.

[44] Muangpat P., Yooyangket T., Fukruksa C., Suwannaroj M., Yimthin T., Sitthisak S., Chantratita N., Vitta A., Tobias N.J., Bode H.B. (2017). Screening of the Antimicrobial Activity against Drug Resistant Bacteria of *Photorhabdus* and *Xenorhabdus* Associated with Entomopathogenic Nematodes from Mae Wong National Park, Thailand. Front. Microbiol..

[45] Seier-Petersen M.A., Jasni A., Aarestrup F.M., Vigre H., Mullany P., Roberts A., Agersø Y. (2014). Effect of Subinhibitory Concentrations of Four Commonly Used Biocides on the Conjugative Transfer of Tn 916 in *Bacillus subtilis*. J. Antimicrob. Chemother..

[46] Torrungruang K., Vichienroj P., Chutimaworapan S. (2007). Antibacterial Activity of Mangosteen Pericarp Extract against Cariogenic *Streptococcus mutans*. Chulalongkorn Univ. Dent. J..

[47] Richter M., Rosselló-Móra R. (2009). Shifting the Genomic Gold Standard for the Prokaryotic Species Definition. Proc. Natl. Acad. Sci. USA.

[48] Awori R.M., Waturu C.N., Pidot S.J., Amugune N.O., Bode H.B. (2023). Draft Genomes, Phylogenomic Reconstruction and Comparative Genome Analysis of Three *Xenorhabdus* Strains Isolated from Soil-Dwelling Nematodes in Kenya. Access Microbiol..

[49] Chaston J.M., Suen G., Tucker S.L., Andersen A.W., Bhasin A., Bode E., Bode H.B., Brachmann A.O., Cowles C.E., Cowles K.N. (2011). The Entomopathogenic Bacterial Endosymbionts *Xenorhabdus* and *Photorhabdus*: Convergent Lifestyles from Divergent Genomes. PLoS ONE.

[50] Ogier J.-C., Pagès S., Bisch G., Chiapello H., Médigue C., Rouy Z., Teyssier C., Vincent S., Tailliez P., Givaudan A. (2014). Attenuated Virulence and Genomic Reductive Evolution in the Entomopathogenic Bacterial Symbiont Species, *Xenorhabdus poinarii*. Genome Biol. Evol..

[51] Barona-Gómez F., Wong U., Giannakopulos A.E., Derrick P.J., Challis G.L. (2004). Identification of a Cluster of Genes That Directs Desferrioxamine Biosynthesis in *Streptomyces coelicolor* M145. J. Am. Chem. Soc..

[52] Stow P.R., Reitz Z.L., Johnstone T.C., Butler A. (2021). Genomics-Driven Discovery of Chiral Triscatechol Siderophores with Enantiomeric Fe (Iii) Coordination. Chem. Sci..

[53] Grammbitter G.L., Schmalhofer M., Karimi K., Shi Y.-M., Schöner T.A., Tobias N.J., Morgner N., Groll M., Bode H.B. (2019). An Uncommon Type II PKS Catalyzes Biosynthesis of Aryl Polyene Pigments. J. Am. Chem. Soc..

[54] Zhang S.-D., Isbrandt T., Lindqvist L.L., Larsen T.O., Gram L. (2021). Holomycin, an Antibiotic Secondary Metabolite, Is Required for Biofilm Formation by the Native Producer *Photobacterium galatheae* S2753. Appl. Environ. Microbiol..

[55] Fu J., Bian X., Hu S., Wang H., Huang F., Seibert P.M., Plaza A., Xia L., Müller R., Stewart A.F. (2012). Full-Length RecE Enhances Linear-Linear Homologous Recombination and Facilitates Direct Cloning for Bioprospecting. Nat. Biotechnol..

[56] Wang G., Zhao Z., Ke J., Engel Y., Shi Y.-M., Robinson D., Bingol K., Zhang Z., Bowen B., Louie K. (2019). CRAGE Enables Rapid Activation of Biosynthetic Gene Clusters in Undomesticated Bacteria. Nat. Microbiol..

[57] Bode H.B., Brachmann A.O., Jadhav K.B., Seyfarth L., Dauth C., Fuchs S.W., Kaiser M., Waterfield N.R., Sack H., Heinemann S.H. (2015). Structure Elucidation and Activity of Kolossin A, the D-/L-Pentadecapeptide Product of a Giant Nonribosomal Peptide Synthetase. Angew. Chem. Int. Ed. Engl..

[58] Hirschmann M., Grundmann F., Bode H.B. (2017). Identification and Occurrence of the Hydroxamate Siderophores Aerobactin, Putrebactin, Avaroferrin and Ochrobactin C as Virulence Factors from Entomopathogenic Bacteria. Environ. Microbiol..

[59] Li J.-H., Cho W., Hamchand R., Oh J., Crawford J.M. (2021). A Conserved Nonribosomal Peptide Synthetase in *Xenorhabdus bovienii* Produces Citrulline-Functionalized Lipopeptides. J. Nat. Prod..

[60] Crawford J.M., Kontnik R., Clardy J. (2010). Regulating Alternative Lifestyles in Entomopathogenic Bacteria. Curr. Biol..

[61] Tamang P., Upadhaya A., Paudel P., Meepagala K., Cantrell C.L. (2024). Mining Biosynthetic Gene Clusters of Pseudomonas Vancouverensis Utilizing Whole Genome Sequencing. Microorganisms.

[62] Hou Z., Zhou Y., Li J., Zhang X., Shi X., Xue X., Li Z., Ma B., Wang Y., Li M. (2015). Selective in Vivo and in Vitro Activities of 3, 3′-4-Nitrobenzylidene-Bis-4-Hydroxycoumarin against Methicillin-Resistant *Staphylococcus aureus* by Inhibition of DNA Polymerase III. Sci. Rep..

[63] Phitaktim S., Chomnawang M., Sirichaiwetchakoon K., Dunkhunthod B., Hobbs G., Eumkeb G. (2016). Synergism and the Mechanism of Action of the Combination of α-Mangostin Isolated from *Garcinia mangostana* L. and Oxacillin against an Oxacillin-Resistant *Staphylococcus saprophyticus*. BMC Microbiol..

