# Supplementary material for: Biosynthetic Gene Cluster Mining and In Vitro Evaluation of the Antimicrobial and Cytotoxic Potential of Xenorhabdus thuongxuanensis
Source: Biology (Basel). 2026 Sep 28;15(19):1716. doi: 10.3390/biology15191716 (PMC13663482; doi:10.3390/biology15191716)
Supplement: Supplementary file 1 [file biology-15-01716-s001.zip › Figure S1.pdf]

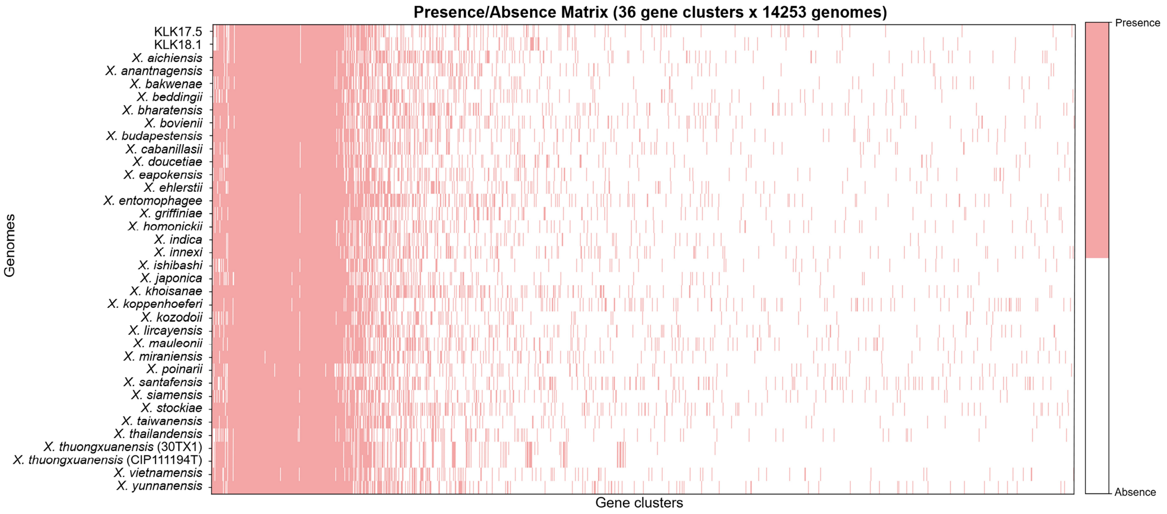


**Figure** S1: Gene presence–absence profile across 36 *Xenorhabdus* genomes. The plot shows the distribution of genes across 36 *Xenorhabdus* genomes. Light red indicates gene presence in the corresponding genome, whereas white indicates gene absence
