# Supplementary material for: Biosynthetic Gene Cluster Mining and In Vitro Evaluation of the Antimicrobial and Cytotoxic Potential of Xenorhabdus thuongxuanensis
Source: Biology (Basel). 2026 Sep 28;15(19):1716. doi: 10.3390/biology15191716 (PMC13663482; doi:10.3390/biology15191716)
Supplement: Supplementary file 1 [file biology-15-01716-s001.zip › Figure S3.pdf]

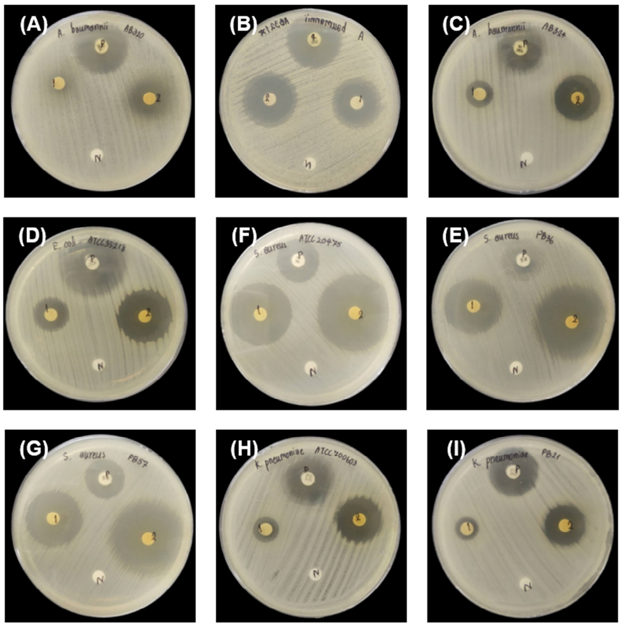

Figure S3: Antibacterial activity of crude extracts from *X. thuongxuanensis* KLK17.5 (1) and *X. thuongxuanensis* KLK18.1 (1), compared with DMSO (N) and an antibiotic disc (P), against pathogenic bacteria including *A. baumannii* AB320 (XDR; A), *A. baumannii* AB321 (MDR; B), *A. baumannii* AB324 (XDR; C), *E. coli* ATCC35218 (ESBL; D), *S. aureus* ATCC20475 (MRSA; E), *S. aureus* PB36 (MRSA; F), *S. aureus* PB57 (MRSA; G), , *K. pneumoniae* ATCC700603 (MDR; H), and *K. pneumoniae* PB21 (ESBL+CRE; I).
