# Supplementary material for: Biosynthetic Gene Cluster Mining and In Vitro Evaluation of the Antimicrobial and Cytotoxic Potential of Xenorhabdus thuongxuanensis
Source: Biology (Basel). 2026 Sep 28;15(19):1716. doi: 10.3390/biology15191716 (PMC13663482; doi:10.3390/biology15191716)
Supplement: Supplementary file 1 [file biology-15-01716-s001.zip › Table S10.pdf]

Table S10. Predicted biosynthetic gene clusters (BGCs) in *X. thuongxuanensis* 30TX1 (reference strain) identified using antiSMASH.

Region	Cluster	Type	Compound prediction	Organism	Approach	MiBig/References	BLASTp		
							Identity (%)	Query coverage (%)	E-value
region 1.1	1	azole-containing-RiPP	unknown	NA	manaul	NA	NA	NA	NA
region 1.2	2	NI-siderophore	desferrioxamine E	*Pantoea agglomerans*	manual	BGC0001572	53.72%	98%	0
region 3.1	3	NRPS	trichrysobactin cyclic/ trichrysobactin/ chrysobactin/ dichrysobactin	*Dickeya chrysanthemi*	antiSMASH (medium)	BGC0002414	61.02%	92%	0
region 5.1	4	hybrid: NRPS,T1PKS	unknown	NA	manaul	NA	NA	NA	NA
region 6.1	5	aryl polyene	aryl polyenes	*Xenorhabdus doucetiae*	antiSMASH (high)	BGC0002008	NA	NA	NA
region 8.1	6	NRPS	holomycin	*Photobacterium galatheae*	antiSMASH (medium)	BGC0002412	56.12%	100%	0
region 9.1	7	hybrid: NRPS,T1PKS	unknown	NA	manaul	NA	NA	NA	NA
region 10.1	8	NRPS	GameXPeptide C	*Photorhabdus laumondii* subsp. *laumondii* TTO1	manual	BGC0001128	81.14%	100%	0
	9	hybrid: NRPS,NRPS-like	unknown	NA	manaul	NA	NA	NA	NA
region 12.1	10	NRPS	unknown	NA	manaul	NA	NA	NA	NA
region 13.1	11	T1PKS	unknown	NA	manaul	NA	NA	NA	NA
region 22.1	12	hybrid: NRPS,RiPP-like	unknown	NA	manaul	NA	NA	NA	NA

region 23.1	13	hybrid: NRPS,PKS	HTTPCA/ prepiscibactin/ piscibactin	*Photorhabdus laumondii* subsp. *laumondii*	manual	BGC0002715	85.19%	100%	0
region 26.1	14	NRPS	unknown	NA	manaul	NA	NA	NA	NA
region 29.1	15	NRPS	kolossin	*Photorhabdus laumondii* subsp. *laumondii* TTO1	antiSMASH (high)	BGC0001641	NA	NA	NA
region 33.1	16	NI-siderophore	putrebactin, avaroferrin	*Xenorhabdus budapestensis*	antiSMASH (high)	BGC0001870	NA	NA	NA
region 41.1	17	NRPS	unknown	NA	manaul	NA	NA	NA	NA
region 45.1	18	betalactone	IOC	NA	manaul	Shi et al., 2022	NA	NA	NA
region 56.1	19	terpene-precursor	unknown	NA	manaul	NA	NA	NA	NA
region 56.2	20	phosphonate	unknown	NA	manaul	NA	NA	NA	NA
region 67.1	21	NRPS-like	xenematide	*Xenorhabdus nematophila* AN6/1	antiSMASH (high)	BGC0001825	NA	NA	NA
region 78.1	22	betalactone	unknown	NA	manaul	NA	NA	NA	NA
region 78.2	23	NRPS	bovienimide A	*Xenorhabdus bovienii* SS-2004	antiSMASH (high)	BGC0002135	NA	NA	NA

NA = not applicable
