# Supplementary material for: Biosynthetic Gene Cluster Mining and In Vitro Evaluation of the Antimicrobial and Cytotoxic Potential of Xenorhabdus thuongxuanensis
Source: Biology (Basel). 2026 Sep 28;15(19):1716. doi: 10.3390/biology15191716 (PMC13663482; doi:10.3390/biology15191716)
Supplement: Supplementary file 1 [file biology-15-01716-s001.zip › Table S11.pdf]

Table S11. Predicted biosynthetic gene clusters (BGCs) in *X. thuongxuanensis* CIP111194^{T} (type strain) identified using antiSMASH.

**Region**	**Cluster**	**Type**	**Compound prediction**	**Organism**	**Approach**	**MiBig/References**	**BLASTp**		
							Identity (%)	Query coverage (%)	E-value
region 1.1	1	terpene-precursor	unknown	NA	manaul	NA	NA	NA	NA
region 1.2	2	phosphonate	unknown	NA	manaul	NA	NA	NA	NA
region 3.1	3	NRPS	trichrysobactin cyclic/ trichrysobactin/ chrysobactin/ dichrysobactin	*Dickeya chrysanthemi*	antiSMASH (medium)	BGC0002414	67.47%	100%	0
region 4.1	4	NRPS	unknown	NA	manaul	NA	NA	NA	NA
region 5.1	5	azole-containing-RiPP	unknown	NA	manaul	NA	NA	NA	NA
region 6.1	6	hybrid: NRPS,T1PKS	unknown	NA	manaul	NA	NA	NA	NA
region 8.1	7	NI-siderophore	desferrioxamine E	*Pantoea agglomerans*	manual	BGC0001572	53.72%	99%	0
region 11.1	8	NRPS	bovienimide A	*Xenorhabdus bovienii SS-2004*	antiSMASH (high)	BGC0002135	NA	NA	NA
region 12.1	9	hybrid: NRPS,T1PKS	unknown	NA	manaul	NA	NA	NA	NA
region 14.1	10	betalactone	IOC	NA	manaul	Shi et al., 2022	NA	NA	NA
region 15.1	11	NRPS	xenoamicin A-B	*Xenorhabdus doucetiae*	manual	BGC0000464	77.26%	92%	0
region 20.1	12	NRPS	GameXPeptide C	*Photorhabdus laumondii* subsp. *laumondii* TTO1	manual	BGC0001128	81.14%	100%	0
	13	hybrid: NRPS,NRPS-like	unknown	NA	manaul	NA	NA	NA	NA

region 22.1	14	aryl polyene	aryl polyenes	*Xenorhabdus doucetiae*	antiSMASH (high)	BGC0002008	NA	NA	NA
region 24.1	15	betalactone	unknown	NA	manaul	NA	NA	NA	NA
region 26.1	16	hybrid: NRPS,PKS	HTTPCA/ prepiscibactin/ piscibactin	*Photorhabdus laumondii* subsp. *laumondii*	manual	BGC0002715	81%	99%	0
region 27.1	17	T1PKS	unknown	NA	manaul	NA	NA	NA	NA
region 32.1	18	NRPS	holomycin	*Photobacterium galatheae*	antiSMASH (medium)	BGC0002412	56%	100%	0
region 65.1	19	NI-siderophore	putrebactin, avaroferrin	*Xenorhabdus budapestensis*	antiSMASH (high)	BGC0001870	NA	NA	NA

NA = not applicable
