# Supplementary material for: Biosynthetic Gene Cluster Mining and In Vitro Evaluation of the Antimicrobial and Cytotoxic Potential of Xenorhabdus thuongxuanensis
Source: Biology (Basel). 2026 Sep 28;15(19):1716. doi: 10.3390/biology15191716 (PMC13663482; doi:10.3390/biology15191716)
Supplement: Supplementary file 1 [file biology-15-01716-s001.zip › Table S12.pdf]

Table S12. Comparison of annotated features of predicted biosynthetic gene clusters across four *Xenorhabdus thuongxuanensis* strains.

Type BGCs	Compound similar	Strains	Core biosynthetic genes	Tailoring enzymes	Regulators	Transporters
NI-siderophore	desferrioxamine E	30TX1	1 core gene (JHDOGEKL_00292: hypothetical protein)	oxygenase (1); JHDOGEKL_00293: lysine/ornithine N-monooxygenase	not identified	not identified
		CIP111194ᵀ	1 core gene (FCLFDAEH_00719: hypothetical protein)	oxygenase (1); FCLFDAEH_00718: lysine/ornithine N-monooxygenase	not identified	not identified
NRPS	trichrysobactin cyclic/ trichrysobactin/ chrysobactin/ dichrysobactin	30TX1	3 core genes (JHDOGEKL_00469: 2,3-dihydro-2,3-dihydroxybenzoate dehydrogenase, JHDOGEKL_00472: Isochorismate synthase EntC, and JHDOGEKL_00476: linear gramicidin synthase subunit D)	6 genes: dehydrogenase (1); JHDOGEKL_00469: short-chain dehydrogenase/reductase SDR, esterase hydrolases (2); JHDOGEKL_00470: isochorismatase, and JHDOGEKL_00474: enterobactin/ferric enterobactin esterase, isomerase (1); JHDOGEKL_00472: isochorismate synthase , and ligases (2); JHDOGEKL_00471: AMP-dependent synthetase and ligase, and JHDOGEKL_00476: condensation domain-containing protein	not identified	2 transporters (JHDOGEKL_00473: Ferrichrome outer membrane transporter/phage receptor, and JHDOGEKL_00477: Enterobactin exporter EntS)
		CIP111194ᵀ	3 core genes (FCLFDAEH_00322: 2,3-dihydro-2,3-dihydroxybenzoate dehydrogenase, FCLFDAEH_00325:	6 genes: dehydrogenase (1); FCLFDAEH_00322: short-chain dehydrogenase/reductase SDR, esterase hydrolases (2); FCLFDAEH_00323: isochorismatase , and	not identified	2 transporters (FCLFDAEH_00326: Ferrichrome outer membrane transporter/phage receptor, and

			Isochorismate synthase EntC, and FCLFDAEH_00329: linear gramicidin synthase subunit D)	FCLFDAEH_00327: enterobactin/ferric enterobactin esterase, isomerase (1); FCLFDAEH_00325: isochorismate synthase , and ligases (2); FCLFDAEH_00324: AMP-dependent synthetase and ligase, and FCLFDAEH_00329: condensation domain-containing protein		FCLFDAEH_00330: Enterobactin exporter EntS)
		KLK17.5	3 core genes (KLK17.5_00425: ,3-dihydro-2,3-dihydroxybenzoate dehydrogenase, KLK17.5_00428: Isochorismate synthase EntC and KLK17.5_00432: linear gramicidin synthase subunit D)	6 genes: dehydrogenase (1); KLK17.5_00425: short-chain dehydrogenase/reductase SDR, esterase hydrolases (2); KLK17.5_00426: isochorismatase, KLK17.5_00430: enterobactin/ferric enterobactin esterase, isomerase (1); KLK17.5_00428: isochorismate synthase , and ligases (2); KLK17.5_00427: AMP-dependent synthetase and ligase and KLK17.5_00432: condensation domain-containing protein	not identified	2 transporters (KLK17.5_00429: Ferrichrome outer membrane transporter/phage receptor and KLK17.5_00433: Enterobactin exporter EntS)
arylpolyene	aryl polyenes	30TX1	2 core genes (JHDOGEKL_00689: 3-oxoacyl-[acyl-carrier-protein] synthase 1, and JHDOGEKL_00692: hypothetical protein)	7 genes: dehydrogenase (1); JHDOGEKL_00690: short-chain dehydrogenase/reductase SDR , acyltransferase (1); JHDOGEKL_00703: acyltransferase , methyltransferase (1); JHDOGEKL_00705: O-methyltransferase, and ligases (4); JHDOGEKL_00689 and	not identified	not identified

				JHDOGEKL_00692: Beta-ketoacyl synthase, JHDOGEKL_00697: polyprenol-monophosphomannose synthase ppm1, and JHDOGEKL_00699: AMP-dependent synthetase and ligase		
		CIP111194T	2 core genes (FCLFDAEH_01599: 3-oxoacyl-[acyl-carrier-protein] synthase 1, and FCLFDAEH_01602: hypothetical protein)	7 genes: dehydrogenase (1); FCLFDAEH_01600: short-chain dehydrogenase/reductase SDR, acyltransferase (1); FCLFDAEH_01613: acyltransferas e , methyltransferase (1); FCLFDAEH_01615: O-methyltransferase, and ligases (4); FCLFDAEH_01599 and FCLFDAEH_01602: beta-ketoacyl synthase, FCLFDAEH_01607: polyprenol-monophosphomannose synthase ppm1, and FCLFDAEH_01609: AMP-dependent synthetase and ligase	not identified	not identified
		KLK17.5	1 core gene (KLK17.5_01641: 3-oxoacyl-[acyl-carrier-protein] synthase 1)	5 genes: acetyltransferase (1); KLK17.5_01668: acyltransferase , methyltransferase (1); KLK17.5_01670: O-methyltransferase, and ligases (3); KLK17.5_01641: beta-ketoacyl synthase, KLK17.5_01662: polyprenol-monophosphomannose synthase ppm1, and KLK17.5_01664: AMP-dependent synthetase and ligase	not identified	not identified

		KLK18.1	2 core gene (KLK18.1_02883: hypothetical protein and KLK18.1_02886: 3-oxoacyl-[acyl-carrier-protein] synthase 1)	7 genes: dehydrogenase (1); KLK18.1_02885: short-chain dehydrogenase/reductase SDR, acyltransferase (1); KLK18.1_02872: acyltransferase, methyltransferase (1); KLK18.1_02870: O-methyltransferase, and ligases (4); KLK18.1_02876: AMP-dependent synthetase and ligase, KLK18.1_02878: polyprenol-monophosphomannose synthase ppm1, KLK18.1_02883 and KLK18.1_02886: Beta-ketoacyl synthase	not identified	not identified
NRPS	holomycin	30TX1	1 core gene (JHDOGEKL_00925: Phenyloxazoline synthase MbtB)	3 genes: dehydrogenase (1); JHDOGEKL_00922: acyl-CoA dehydrogenase, esterase (1); JHDOGEKL_00923: thioesterase , ligase (1); JHDOGEKL_00925: condensation domain-containing protein	not identified	not identified
		CIP111194[T]	1 core gene (FCLFDAEH_02110: Phenyloxazoline synthase MbtB)	3 genes: dehydrogenase (1); FCLFDAEH_02107: acyl-CoA dehydrogenase, esterase (1); FCLFDAEH_02108: thioesterase, ligase (1); FCLFDAEH_02110: condensation domain-containing protein	not identified	not identified
		KLK17.5	1 core gene (KLK17.5_00575: Phenyloxazoline synthase MbtB)	3 genes: dehydrogenase (1); KLK17.5_00572: acyl-CoA dehydrogenase, esterase (1); KLK17.5_00573: thioesterase,	not identified	1 transporter (KLK17.5_00577: Putative multidrug

				ligase (1); KLK17.5_00575: condensation domain-containing protein		resistance protein MdtD)
		KLK18.1	1 core gene (KLK18.1_00574: Phenyloxazoline synthase MbtB)	3 genes: dehydrogenase (1); KLK18.1_00571:acyl-CoA dehydrogenase , esterase (1); KLK18.1_00572:thioesterase, ligase (1); KLK18.1_00574:condensation domain-containing protein	not identified	1 transporter (KLK18.1_00578: Putative multidrug resistance protein MdtD)
NRPS	GameXPeptide C	30TX1	1 core gene (JHDOGEKL_01016: Tyrocidine synthase 3)	ligase (1); JHDOGEKL_01016: condensation domain-containing protein	not identified	not identified
		CIP111194[T]	1 core gene (FCLFDAEH_01513: Tyrocidine synthase 3)	ligase (1); FCLFDAEH_01513: condensation domain-containing protein	not identified	not identified
		KLK17.5	1 core gene (KLK17.5_03125: Tyrocidine synthase 3)	ligase (1); KLK17.5_03125: condensation domain-containing protein	not identified	not identified
		KLK18.1	1 core gene (KLK18.1_02972: Tyrocidine synthase 3)	ligase (1); KLK18.1_02972: condensation domain-containing protein	not identified	not identified
NRPS,T1PKS	HTTPCA	30TX1	3 core genes (JHDOGEKL_01926: 2-succinylbenzoate--CoA ligase, JHDOGEKL_01929 and JHDOGEKL_01930: D-alanine--D-alanyl carrier protein ligase)	4 genes: esterase (1); JHDOGEKL_01927: thioesterase and ligases (3); JHDOGEKL_01926 and JHDOGEKL_01930: AMP-dependent synthetase and ligase, and JHDOGEKL_01929: Beta-ketoacyl synthase	1 regulator (JHDOGEKL_01935: HTH-type transcriptional activator RhaR)	4 transporters (JHDOGEKL_01931 and JHDOGEKL_01932: Lipid A export ATP-binding/permease protein MsbA, JHDOGEKL_01933: Anhydromuropeptide permease, and JHDOGEKL_01934: Pesticin receptor)

		CIP111194[T]	3 core genes (FCLFDAEH_01851 and FCLFDAEH_01852: D-alanine--D-alanyl carrier protein ligase, and FCLFDAEH_01855: 2-succinylbenzoate--CoA ligase)	4 genes: esterase (1); FCLFDAEH_01854: thioesterase and ligases (3); FCLFDAEH_01851 and FCLFDAEH_01855: AMP-dependent synthetase and ligase, and FCLFDAEH_01852: Beta-ketoacyl synthase	1 regulator (FCLFDAEH_01846: HTH-type transcriptional activator RhaR)	4 transporters (FCLFDAEH_01847: Pesticin receptor, FCLFDAEH_01848: Anhydromuropeptide permease, FCLFDAEH_01849 and FCLFDAEH_01850: Lipid A export ATP-binding/permease protein MsbA)
		KLK17.5	3 core genes (KLK17.5_02868: 2-succinylbenzoate--CoA ligase, KLK17.5_02871 and KLK17.5_02872; D-alanine--D-alanyl carrier protein ligase)	4 genes: esterase (1); KLK17.5_02869: thioesterase and ligases (3); KLK17.5_02868: AMP-dependent synthetase and ligase, KLK17.5_02871: Beta-ketoacyl synthase, and KLK17.5_02872: AMP-dependent synthetase and ligase	1 regulator (KLK17.5_02877: HTH-type transcriptional activator RhaR)	4 transporters (KLK17.5_02873 and KLK17.5_02874: Lipid A export ATP-binding/permease protein MsbA, KLK17.5_02875: Anhydromuropeptide permease, and KLK17.5_02876: Pesticin receptor)
		KLK18.1	3 core genes (KLK18.1_01756 and KLK18.1_01757: D-alanine--D-alanyl carrier protein ligase and KLK18.1_01760: 2-succinylbenzoate--CoA ligase)	4 genes: esterase hydrolase (1); KLK18.1_01759: thioesterase and ligases (3); KLK18.1_01756 and KLK18.1_01760: AMP-dependent synthetase and ligase and KLK18.1_01757: Beta-ketoacyl synthase	1 regulator (KLK18.1_01751: HTH-type transcriptional activator RhaR)	4 transporters (KLK18.1_01752: Pesticin receptor, KLK18.1_01753: Anhydromuropeptide permease, KLK18.1_01754 and KLK18.1_01755: Lipid A export ATP-binding/permease protein MsbA)

NRPS	kolossin	30TX1	1 core gene (JHDOGEKL_02276: Tyrocidine synthase 3)	ligase (1); JHDOGEKL_02276: condensation domain-containing protein	not identified	not identified
NI-siderophore	putrebactin, avaroferrin	30TX1	1 core gene (JHDOGEKL_02346: Aerobactin synthase)	oxygenase (1); JHDOGEKL_02354: lysine/ornithine N-monooxygenase	not identified	5 transporters (JHDOGEKL_02347: FhuE receptor, JHDOGEKL_02348: hypothetical protein, JHDOGEKL_02349: Hemin transport system permease protein HmuU, JHDOGEKL_02350: Petrobactin import system permease protein FpuB, and JHDOGEKL_02351: putative siderophore transport system ATP-binding protein YusV)
		CIP111194[T]	1 core gene (FCLFDAEH_02913: Aerobactin synthase)	oxygenase (1); FCLFDAEH_02905: lysine/ornithine N-monooxygenase	not identified	5 transporters (FCLFDAEH_02908: putative siderophore transport system ATP-binding protein YusV, FCLFDAEH_02909: Petrobactin import system permease protein FpuB, FCLFDAEH_02910: Hemin transport system permease

						protein HmuU, FCLFDAEH_02910: Hemin transport system permease protein HmuU, FCLFDAEH_02911: hypothetical protein, and FCLFDAEH_02912: FhuE receptor)
		KLK17.5	2 core genes (KLK17.5_00957: Aerobactin synthase and KLK17.5_00966: hypothetical protein)	2 genes: oxygenase (1); KLK17.5_00965: lysine/ornithine N-monooxygenase, and decarboxylase (1); KLK17.5_00964: Decarboxylase, pyridoxal-dependent	not identified	5 transporters (KLK17.5_00958: FhuE receptor, KLK17.5_00959: hypothetical protein, KLK17.5_00960: putative siderophore transport system permease protein YfiZ, KLK17.5_00961: Petrobactin import system permease protein FpuB and KLK17.5_00962: putative siderophore transport system ATP-binding protein YusV)
		KLK18.1	2 core genes (KLK18.1_00966: Aerobactin synthase, KLK18.1_00975: hypothetical protein)	2 genes: oxygenase (1); KLK18.1_00974: lysine/ornithine N-monooxygenase , and decarboxylase (1); KLK18.1_00973: Decarboxylase, pyridoxal-dependent	not identified	5 transporters (KLK18.1_00967: FhuE receptor, KLK18.1_00968: Vitamin B12-binding protein,

						KLK18.1_00969: Hemin transport system permease protein HmuU, KLK18.1_00970: Petrobactin import system permease protein FpuB, KLK18.1_00971: putative siderophore transport system ATP-binding protein YusV)
betalactone	IOC	30TX1	1 core gene (JHDOGEKL_02764 :Long-chain-fatty-acid--CoA ligase FadD15)	not identified	1 regulatory gene (JHDOGEKL_02763 : HTH-type transcriptional regulator LeuO)	not identified
		CIP111194[T]	1 core gene (FCLFDAEH_01191:Long-chain-fatty-acid--CoA ligase FadD15)	not identified	1 regulatory gene (FCLFDAEH_01192: HTH-type transcriptional regulator LeuO)	not identified
		KLK17.5	1 core gene (KLK17.5_01993 :Long-chain-fatty-acid--CoA ligase FadD15)	not identified	1 regulatory gene (KLK17.5_01992 : HTH-type transcriptional regulator LeuO)	not identified
		KLK18.1	1 core gene (KLK18.1_02567:Long-chain-fatty-acid--CoA ligase FadD15)	ligase (1); KLK18.1_02567: AMP-dependent synthetase and ligase	1 regulatory gene (KLK18.1_02568: HTH-type transcriptional regulator LeuO)	not identified

NRPS-like	xenematide	30TX1	1 core gene (JHDOGEKL_03061: -alanine--D-alanyl carrier protein ligase)	ligase (1); JHDOGEKL_03061: AMP-dependent synthetase and ligase	not identified	not identified
NRPS	xenoamicin A-B	CIP111194[T]	4 core genes (FCLFDAEH_01225, FCLFDAEH_01227, and FCLFDAEH_01229: Tyrocidine synthase 3, FCLFDAEH_01226, and Gramicidin S synthase 2	7 genes: dehydrogenase (1); FCLFDAEH_01231: alcohol dehydrogenase, oxidoreductase (1); FCLFDAEH_01230: oxidoreductas e, acyltransferase (2); FCLFDAEH_01225 and FCLFDAEH_01229: polyketide synthase associated protein papA3 , decarboxylase (1); FCLFDAEH_01228: Decarboxylase , pyridoxal-dependent , and ligases (2); FCLFDAEH_01226 and FCLFDAEH_01227: condensation domain-containing protein	not identified	not identified
		KLK17.5	5 core genes (KLK17.5_01447, KLK17.5_01448, KLK17.5_01450 and KLK17.5_01452: Tyrocidine synthase 3, and KLK17.5_01451: Gramicidin S synthase 2)	9 genes: dehydrogenase (1); KLK17.5_01445: alcohol dehydrogenase, oxidoreductase (1); KLK17.5_01446: oxidoreductase, acyltransferase (2); KLK17.5_01447 and KLK17.5_01452: polyketide synthase associated protein papA3, decarboxylase (1); KLK17.5_01449: Decarboxylase, pyridoxal-dependent , ligases (3); KLK17.5_01448, KLK17.5_01450, and KLK17.5_01451: condensation domain-containing protein, and permease (1);	not identified	not identified

				KLK17.5_01457: phenylalanine-specific permease		
		KLK18.1	4 core genes (KLK18.1_01446 and KLK18.1_01447: Tyrocidine synthase 3, KLK18.1_01448 and KLK18.1_01449: Linear gramicidin synthase subunit D)	8 genes: dehydrogenase (1); KLK18.1_01444: alcohol dehydrogenase, oxidoreductase (1); KLK18.1_01445: oxidoreductase, acyltransferase (1): KLK18.1_01446: polyketide synthase associated protein papA3, decarboxylase (1); KLK18.1_01450: Decarboxylase, pyridoxal-dependent, ligases (3); KLK18.1_01447 and KLK18.1_01449: condensation domain-containing protei, KLK18.1_01448: AMP-dependent synthetase and ligase, and permease (1); KLK18.1_01458: phenylalanine-specific permease	not identified	not identified
NRPS	rhabduscin	KLK17.5	not identified	2 genes: oxygenase (1); KLK17.5_02320: dioxygenase, TauD/TfdA, and glycosyltransferase sulfotransferase (1); KLK17.5_02319: glycosyltransferase, MGT family	not identified	not identified
NRPS	bovienimide A	30TX1	1 core gene (JHDOGEKL_03336: Tyrocidine synthase 3)	ligase (1); JHDOGEKL_03336: AMP-dependent synthetase and ligase	not identified	not identified
		CIP111194[T]	1 core gene (FCLFDAEH_01012: Tyrocidine synthase 3)	ligase (1); FCLFDAEH_01012: AMP-dependent synthetase and ligase	not identified	not identified

		KLK18.1	1 core gene (KLK18.1_01410: Tyrocidine synthase 3)	ligase (1); KLK18.1_01410: AMP-dependent synthetase and ligase	not identified	not identified
