# Supplementary material for: Biosynthetic Gene Cluster Mining and In Vitro Evaluation of the Antimicrobial and Cytotoxic Potential of Xenorhabdus thuongxuanensis
Source: Biology (Basel). 2026 Sep 28;15(19):1716. doi: 10.3390/biology15191716 (PMC13663482; doi:10.3390/biology15191716)
Supplement: Supplementary file 1 [file biology-15-01716-s001.zip › Table S13.pdf]

Table S13. Antibacterial activity of crude extracts from *X. thuongxuanensis* against drug-resistant bacteria using disc diffusion.

Code	Disc diffusion of drug-resistant bacterial (mm)														
	***A. baumannii* AB320 (XDR)**	***A. baumannii* AB321 (MDR)**	***A. baumannii* AB322 (MDR)**	***A. baumannii* AB324 (XDR)**	***S. aureus* ATCC 20475 (MRSA)**	***S. aureus* PB36 (MRSA)**	***S. aureus* PB57 (MRSA)**	***E. coli* ATCC 35218 (ESBL)**	***E. coli* PB1 (ESBL+MDR)**	***E. coli* PB30 (MDR)**	***E. coli* PB23 (ESBL+CRE)**	***K. pneumoniae* ATCC 700603 (ESBL)**	***K. pneumoniae* PB5 (ESBL+MDR)**	***K. pneumoniae* PB21 (ESBL+CRE)**	***P. aeruginosa* ATCC27853 (MDR)**
KLK17.5	6	29	6	13	30	27	28	14	16	6	16	13	27	12	13
KLK18.1	19	34	10	23	36	33	34	28	27	15	27	22	33	22	30.5
Positive	19	32	22	22	25	25	20	28	6	6	6	18	6	25	24
