# Supplementary material for: Biosynthetic Gene Cluster Mining and In Vitro Evaluation of the Antimicrobial and Cytotoxic Potential of Xenorhabdus thuongxuanensis
Source: Biology (Basel). 2026 Sep 28;15(19):1716. doi: 10.3390/biology15191716 (PMC13663482; doi:10.3390/biology15191716)
Supplement: Supplementary file 1 [file biology-15-01716-s001.zip › Table S14.pdf]

Table S14. Antibacterial activity of crude extracts from *X. thuongxuanensis* against drug-resistant bacteria using broth dilution assay.

Code	Broth dilution assay of drug-resistant bacteria													
	Acinetobacter baumannii								*Staphylococcus aureus*					
	**AB320**		**AB321**		**AB322**		**AB324**		**ATCC20475**		**PB36**		**PB57**	
	**MIC**	**MBC**	**MIC**	**MBC**	**MIC**	**MBC**	**MIC**	**MBC**	**MIC**	**MBC**	**MIC**	**MBC**	**MIC**	**MBC**
KLK17.5	ND	ND	3.13	3.13	ND	ND	6.25	50	0.2	6.25	0.39	3.13	0.39	1.56
KLK18.1	6.25	12.5	3.13	12.5	3.13	12.5	1.56	12.5	0.2	0.78	0.39	0.78	0.2	0.78
DMSO (negative control)	25	100	25	100	25	100	25	100	50	100	50	100	25	100

ND = Not determined.

Table S14. Continue.

Code	Broth dilution assay of drug-resistant bacteria															
	Escherichia coli								*Klebsiella pneumoniae*						*Pseudomonas aeruginosa*	
	**ATCC35218**		**PB1**		**PB30**		**PB231**		**ATCC700603**		**PB5**		**PB21**		**ATCC27853**	
	**MIC**	**MBC**	**MIC**	**MBC**	**MIC**	**MBC**	**MIC**	**MBC**	**MIC**	**MBC**	**MIC**	**MBC**	**MIC**	**MBC**	**MIC**	**MBC**
KLK17.5	0.78	25	3.13	50	ND	ND	3.13	50	6.25	50	0.39	3.13	6.25	50	1.56	3.13
KLK18.1	0.2	3.13	0.39	12.5	6.25	25	0.2	12.5	0.78	25	0.2	0.78	0.39	12.5	0.2	0.39

DMSO (negative control)	25	100	25	100	25	100	25	100	25	100	25	100	25	100	12.5	50

ND = Not determined.
