# Supplementary material for: Biosynthetic Gene Cluster Mining and In Vitro Evaluation of the Antimicrobial and Cytotoxic Potential of Xenorhabdus thuongxuanensis
Source: Biology (Basel). 2026 Sep 28;15(19):1716. doi: 10.3390/biology15191716 (PMC13663482; doi:10.3390/biology15191716)
Supplement: Supplementary file 1 [file biology-15-01716-s001.zip › Table S2.pdf]

Table S2. Genomic characteristics of *X. thuongxuanensis* strains assembled in the current study.

Statistics without reference	*Xenorhabdus thuongxuanensis*			
	**KLK17.5**	**KLK18.1**	**CIP111194^{T}**	**30TX1**
contig	13	12	168	111
contigs (>= 0 bp)	14	17	181	130
contigs (>= 1000 bp)	12	11	142	85
largest contig	1,711,356	3,572,170	157,128	336,111
rRNA	22	22	8	10
repeat_region	3	2	3	3
tRNA	78	81	63	75
tmRNA	1	1	1	1
GC (%)	43.16	42.82	43.21	43.04
N50	1,682,403	3,572,170	61,069	119,455
N90	634,932	3,572,170	13,076	30,508
auN	1,479,774	3,367,756	61,059	144,401
L50	2	1	23	10
L90	3	1	77	33
CDS	3,712	3,424	3,405	3,471
Total length	4,166,903	3,795,363	3,872,110	3,932,016
Total length (>= 0 bp)	4,167,069	3,796,623	3,877,551	3,938,299
Total length (>= 1000 bp)	4,166,190	3,794,567	3,852,773	3,911,800
NCBI accession number	SAMN56417105	SAMN56417106	SAMEA117660565	SAMN05375129
