# Supplementary material for: Biosynthetic Gene Cluster Mining and In Vitro Evaluation of the Antimicrobial and Cytotoxic Potential of Xenorhabdus thuongxuanensis
Source: Biology (Basel). 2026 Sep 28;15(19):1716. doi: 10.3390/biology15191716 (PMC13663482; doi:10.3390/biology15191716)
Supplement: Supplementary file 1 [file biology-15-01716-s001.zip › Table S4.pdf]

Table S4. Pan-genome gene categories across 36 *Xenorhabdus* species genomes

Gene Category	Gene Cluster Count	Percent
accessory	6421.00	45.05
unique	6078.00	42.64
core	1754.00	12.31
