# Supplementary material for: Biosynthetic Gene Cluster Mining and In Vitro Evaluation of the Antimicrobial and Cytotoxic Potential of Xenorhabdus thuongxuanensis
Source: Biology (Basel). 2026 Sep 28;15(19):1716. doi: 10.3390/biology15191716 (PMC13663482; doi:10.3390/biology15191716)
Supplement: Supplementary file 1 [file biology-15-01716-s001.zip › Table S5.pdf]

Table S5. Distribution of Clusters of Orthologous Groups (COG) functional classifications across 36 *Xenorhabdus* species genomes

COG	Description	Count	Percent
J	Translation, ribosomal structure and biogenesis	10722.00	8.45
E	Amino acid transport and metabolism	10394.00	8.19
X	Mobilome: prophages, transposons	9842.00	7.75
M	Cell wall/membrane/envelope biogenesis	9027.00	7.11
K	Transcription	8508.00	6.70
H	Coenzyme transport and metabolism	7518.00	5.92
L	Replication, recombination and repair	6681.00	5.26
G	Carbohydrate transport and metabolism	6591.00	5.19
R	General function prediction only	6445.00	5.08
C	Energy production and conversion	5910.00	4.66
P	Inorganic ion transport and metabolism	5468.00	4.31
I	Lipid transport and metabolism	5127.00	4.04
V	Defense mechanisms	5087.00	4.01
O	Posttranslational modification, protein turnover, chaperones	4939.00	3.89
U	Intracellular trafficking, secretion, and vesicular transport	4825.00	3.80
T	Signal transduction mechanisms	4552.00	3.59
Q	Secondary metabolites biosynthesis, transport and catabolism	3635.00	2.86
F	Nucleotide transport and metabolism	3211.00	2.53
S	Function unknown	3075.00	2.42
N	Cell motility	2448.00	1.93
D	Cell cycle control, cell division, chromosome partitioning	1900.00	1.50
W	Extracellular structures	475.00	0.37
A	RNA processing and modification	229.00	0.18
B	Chromatin structure and dynamics	218.00	0.17
Z	Cytoskeleton	111.00	0.09
