# Supplementary material for: Biosynthetic Gene Cluster Mining and In Vitro Evaluation of the Antimicrobial and Cytotoxic Potential of Xenorhabdus thuongxuanensis
Source: Biology (Basel). 2026 Sep 28;15(19):1716. doi: 10.3390/biology15191716 (PMC13663482; doi:10.3390/biology15191716)
Supplement: Supplementary file 1 [file biology-15-01716-s001.zip › Table S6.pdf]

Table S6. Distribution of COG-annotated pan-genome gene categories across COG functional classifications in 36 *Xenorhabdus* species genomes

COG	Description	COG-annotated pan-genome gene categories					
		**core**	%	**accessory**	%	**unique**	%
J	Translation, ribosomal structure and biogenesis	9,073	12.04	1,728	3.20	87	4.13
A	RNA processing and modification	220	0.29	6	0.01	3	0.14
K	Transcription	4,838	6.42	3,544	6.57	127	6.04
L	Replication, recombination and repair	4,224	5.61	2,513	4.66	127	6.04
B	Chromatin structure and dynamics	182	0.24	35	0.06	1	0.05
D	Cell cycle control, cell division, chromosome partitioning	1,527	2.03	335	0.62	38	1.81
V	Defense mechanisms	1,729	2.29	3,230	5.99	128	6.08
T	Signal transduction mechanisms	3,413	4.53	1,312	2.43	58	2.76
M	Cell wall/membrane/envelope biogenesis	6,417	8.52	2,500	4.64	156	7.41
N	Cell motility	1,919	2.55	541	1.00	24	1.14
Z	Cytoskeleton	110	0.15	0	0.00	1	0.05
W	Extracellular structures	237	0.31	229	0.42	9	0.43
U	Intracellular trafficking, secretion, and vesicular transport	1,908	2.53	2,934	5.44	83	3.94
O	Posttranslational modification, protein turnover, chaperones	3,731	4.95	1,230	2.28	61	2.90

X	Mobilome: prophages, transposons	1,086	1.44	9,690	17.97	187	8.89
C	Energy production and conversion	4,563	6.06	1,556	2.89	68	3.23
G	Carbohydrate transport and metabolism	2,818	3.74	3,968	7.36	109	5.18
E	Amino acid transport and metabolism	7,795	10.34	2,977	5.52	137	6.51
F	Nucleotide transport and metabolism	2,610	3.46	649	1.20	25	1.19
H	Coenzyme transport and metabolism	5,266	6.99	2,305	4.28	93	4.42
I	Lipid transport and metabolism	2,729	3.62	2,495	4.63	147	6.99
P	Inorganic ion transport and metabolism	3,849	5.11	1,644	3.05	67	3.18
Q	Secondary metabolites biosynthesis, transport and catabolism	1,469	1.95	2,844	5.27	119	5.66
R	General function prediction only	2,255	2.99	3,996	7.41	213	10.12
S	Function unknown	1,384	1.84	1,655	3.07	36	1.71
