# Supplementary material for: Biosynthetic Gene Cluster Mining and In Vitro Evaluation of the Antimicrobial and Cytotoxic Potential of Xenorhabdus thuongxuanensis
Source: Biology (Basel). 2026 Sep 28;15(19):1716. doi: 10.3390/biology15191716 (PMC13663482; doi:10.3390/biology15191716)
Supplement: Supplementary file 1 [file biology-15-01716-s001.zip › Table S8.pdf]

Table S8. Predicted biosynthetic gene clusters (BGCs) in *X. thuongxuanensis* KLK17.5 identified using antiSMASH.

Region	Cluster	Type	Compound prediction	Organism	Approach	MiBIG/References	BLASTp		
							Identity (%)	Query coverage (%)	E-value
region 1.1	1	azole-containing-RiPP	unknown	NA	manaul	NA	NA	NA	NA
region 1.2	2	NRPS	trichrysobactin cyclic/ trichrysobactin/ chrysobactin/ dichrysobactin	*Dickeya chrysanthemi*	antiSMASH (medium)	BGC0002414	61.32%	100%	0
	3	hybrid: NRPS,T1PKS	unknown	NA	manaul	NA	NA	NA	NA
region 1.3	4	NRPS	holomycin	*Photobacterium galatheae*	antiSMASH (high)	BGC0002412	NA	NA	NA
region 1.4	5	terpene-precursor	unknown	NA	manaul	NA	NA	NA	NA
region 1.5	6	phosphonate	unknown	NA	manaul	NA	NA	NA	NA
region 1.6	7	NI-siderophore	putrebactin, avaroferrin	*Xenorhabdus budapestensis*	antiSMASH (high)	BGC0001870	NA	NA	NA
region 1.7	8	azole-containing-RiPP	unknown	NA	manaul	NA	NA	NA	NA
region 1.8	9	NRPS	unknown	NA	manaul	NA	NA	NA	NA
region 1.9	10	NRPS	xenoamicin A-B	*Xenorhabdus doucetiae*	antiSMASH (medium)	BGC0000464	77.25%	64%	0
region 2.1	11	aryl polyene	aryl polyenes	*Xenorhabdus doucetiae*	antiSMASH (medium)	BGC0002008	87.14%	61%	0
region 2.2	12	butyrolactone	unknown	NA	manaul	NA	NA	NA	NA
region 2.3	13	betalactone	IOC	NA	manaul	Shi et al., 2022	NA	NA	NA
region 2.4	14	NRPS	rhabduscin	*Xenorhabdus nematophila* ATCC 19061	antiSMASH (high)	BGC0001757	NA	NA	NA

region 2.5	15	NRPS	unknown	NA	manaul	NA	NA	NA	NA
region 2.6	16	hybrid: NRP-metallophore,NRPS, T1PKS	unknown	NA	manaul	NA	NA	NA	NA
	17	hybrid: NRPS,PKS	HTTPCA/ prepiscibactin/ piscibactin	*Photorhabdus laumondii* subsp. *laumondii*	manual	BGC0002715	81%	93%	0
region 2.7	18	hybrid: NRPS,T1PKS	unknown	NA	manaul	NA	NA	NA	NA
region 3.1	19	NRPS	GameXPeptide C	*Photorhabdus laumondii* subsp. *laumondii* TTO1	manual	BGC0001128	78.68%	93%	0
	20	NRPS-like	unknown	NA	manaul	NA	NA	NA	NA
region 4.1	21	NRPS-like	unknown	NA	manaul	NA	NA	NA	NA
region 6.1	22	hybrid: NRPS,T1PKS	unknown	NA	manaul	NA	NA	NA	NA
region 8.1	23	CDPS	unknown	NA	manaul	NA	NA	NA	NA

NA = not applicable
