# Supplementary material for: Biosynthetic Gene Cluster Mining and In Vitro Evaluation of the Antimicrobial and Cytotoxic Potential of Xenorhabdus thuongxuanensis
Source: Biology (Basel). 2026 Sep 28;15(19):1716. doi: 10.3390/biology15191716 (PMC13663482; doi:10.3390/biology15191716)
Supplement: Supplementary file 1 [file biology-15-01716-s001.zip › Table S9.pdf]

Table S9. Predicted biosynthetic gene clusters (BGCs) in *X. thuongxuanensis* KLK18.1 identified using antiSMASH.

**Region**	**Cluster**	**Type**	**Compound prediction**	**Organism**	**Approach**	**MiBig/References**	**BLASTp**		
							Identity (%)	Query coverage (%)	E-value
region 1.1	1	betalactone	unknown	NA	manaul	NA	NA	NA	NA
region 1.2	2	NRPS	holomycin	*Photobacterium galatheae*	antiSMASH (high)	BGC0002412	NA	NA	NA
region 1.3	3	terpene-precursor	unknown	NA	manaul	NA	NA	NA	NA
region 1.4	4	phosphonate	unknown	NA	manaul	NA	NA	NA	NA
region 1.5	5	NI-siderophore	putrebactin, avaroferrin	*Xenorhabdus budapestensis*	antiSMASH (high)	BGC0001870	NA	NA	NA
region 1.6	6	azole-containing-RiPP	unknown	NA	manaul	NA	NA	NA	NA
region 1.7	7	betalactone	unknown	NA	manaul	NA	NA	NA	NA
region 1.8	8	NRPS	bovienimide A	*Xenorhabdus bovienii SS-2004*	manual	BGC0002135	68.99%	100%	0
	9	NRPS	xenoamicin A-B	*Xenorhabdus doucetiae*	manual	BGC0000464	86.72%	100%	0
region 1.9	10	hybrid: NRPS,T1PKS	unknown	NA	manaul	NA	NA	NA	NA
region 1.10	11	hybrid: NRPS,T1PKS	unknown	NA	manaul	NA	NA	NA	NA
region 1.11	12	hybrid: NRPS,PKS	HTTPCA/ prepiscibactin/ piscibactin	*Photorhabdus laumondii* subsp. *laumondii*	manual	BGC0002715	83.86%	100%	0
	13	hybrid: NRP-metallophore,NRPS, RiPP-like,T1PKS	unknown	NA	manaul	NA	NA	NA	NA
region 1.12	14	NRPS	unknown	NA	manaul	NA	NA	NA	NA

region 1.13	15	butyrolactone	unknown	NA	manaul	NA	NA	NA	NA
region 1.14	16	betalactone	IOC	NA	manaul	Shi et al., 2022	NA	NA	NA
region 1.15	17	aryl polyene	aryl polyenes	*Xenorhabdus doucetiae*	antiSMASH (High)	BGC0002008	NA	NA	NA
region 1.16	18	NRPS	unknown	NA	manaul	NA	NA	NA	NA
	19	NRPS	GameXPeptide C	*Photorhabdus laumondii* subsp. *laumondii* TTO1	manual	BGC0001128	68%	100%	0
	20	NRPS-like	unknown	NA	manaul	NA	NA	NA	NA
region 2.1	21	NRPS	unknown	NA	manaul	NA	NA	NA	NA

NA = not applicable
